# Mucus-responsive functionalized emulsions: design, synthesis and study of novel branched polymers as functional emulsifiers†

**DOI:** 10.1039/d0ra05820c

**Published:** 2020-08-18

**Authors:** Stephanie E. Edwards, Sean Flynn, James J. Hobson, Pierre Chambon, Helen Cauldbeck, Steve P. Rannard

**Affiliations:** Department of Chemistry, University of Liverpool Crown Street L69 7ZD UK srannard@liv.ac.uk; Materials Innovation Factory, University of Liverpool Oxford Street Liverpool L7 3NY UK

## Abstract

Mucus lines the moist cavities throughout the body, acting as barrier by protecting the underlying cells against the external environment, but it also hinders the permeation of drugs and drug delivery systems. As the rate of diffusion is low, the development of a system which could increase retention time at the mucosal surface would prove beneficial. Here, we have designed a range of branched copolymers to act as functional mucus-responsive oil-in-water emulsifiers comprising the hydrophilic monomer oligo(ethylene glycol) methacrylate and a hydrophobic dodecyl initiator. The study aimed to investigate the importance of chain end functionality on successful emulsion formation, by systematically replacing a fraction of the hydrophobic chain ends with a secondary poly(ethylene glycol) based hydrophilic initiator in a mixed-initiation strategy; a decrease of up to 75 mole percent of hydrophobic chain ends within the branched polymer emulsifiers was shown to maintain comparative emulsion stability. These redundant chain ends allowed for functionality to be incorporated into the polymers *via* a xanthate based initiator containing a masked thiol group; thiol groups are known to have mucoadhesive character, due to their ability to form disulfide bonds with the cysteine rich areas of mucus. The mucoadhesive nature of emulsions stabilised by thiol-containing branched copolymers was compared to non-functional emulsions in the presence of a biosimilar mucosal substrate and enhanced adherence to the mucosal surface was observed. Importantly, droplet rupture and mucus triggered release of dye-containing oil was seen from previously highly-stable thiol-functional emulsions; this observation was not mirrored by non-functional emulsions where droplet integrity was maintained even in the presence of mucus.

## Abstract 2

Mucoadhesion and mucus-sensitive materials have many applications. Redundant chain-ends within branched polymer emulsifiers have been functionalized with thiols, without compromising emulsion stability, to create mucus-interacting emulsions.

## Graphical abstract

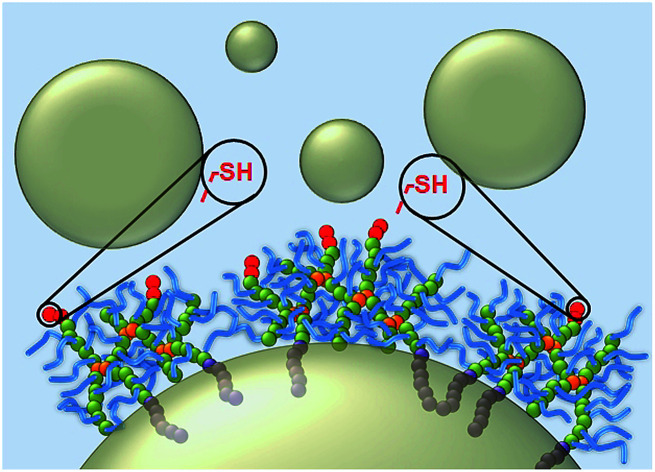

## Introduction

Mucous membranes create the moist exterior of various regions of the body including oral, gastrointestinal (GI), genital and ocular surfaces. For example, mucus is secreted by goblet cells directly onto epithelium cells within the GI tract,^1^ forming a mobile outer layer, that is relatively quickly replaced, and a stationary gel–like layer,^2^ that is adhered to the surface of the goblet cells. Mucosa varies across different anatomical sites with different life-cycles and thicknesses that depend primarily on its specific local function.^3–5^ As a biologically-derived hydrogel^6^ with defined viscoelasticity, rheology and mesh size, mucous membranes act to moderate the passage of exogenous material and prevent infection; in addition, the permeation of many pharmaceutical agents is also restricted in this way. Mucous membranes have, therefore, been a focus for drug delivery over many years and mucoadhesion and mucus permeability have been the subject of much research.^7^

Structurally, mucus comprises a water-swollen network of gel-forming mucins which are cysteine-rich high molecular weight glycosylated proteins. Disulfide bridges between cysteine residues contribute to the physical properties of the mucosal membrane; these sulfur-containing domains offer a chemical target for mucoadhesion and mucopermeation strategies. Mucoadhesion is generally regarded as progressing through a two-step process,^8^ namely contact and consolidation. In situations where adhesion to mucus occurs within a predominantly liquid environment, the contact stage may be considered using conventional DLVO theory, as the resultant contradictory forces of attraction and repulsion must favour a physical interaction; a positive wetting interaction will also favour the contact stage. The material in contact with the mucus may be removed through various shear or stress modes and therefore consolidation of the interaction is required to generate a long-acting adhesion. In a mainly aqueous medium, this may be achieved in several ways^9^ including entrapment, non-covalent intermolecular forces (*e.g.* electrostatic, van der Waals' and hydrogen bonding interactions), and the formation of covalent bonds through chemical reactions (*e.g.* disulfide formation and Michael addition).

Thiol-functional materials and thiol-bearing polymers, often called “thiomers”,^10–12^ have been studied specifically to interact with cysteine residues within mucins, forming disulfide bonds either through thiol/disulfide exchange or thiol–thiol reactions within the mucus network;^13^ evidence for these mechanisms has been established in several reports.^14–17^ To enable drug delivery, thiol-functional polymers have been used to produce tablets,^17^ polymeric micelles^18,19^ and microparticles.^20^ Recently, alkyl-modified carbomers (lightly crosslinked poly(acrylic acid)) were modified with 4-aminothiophenol, l-cysteine or d/l-homocysteine to form a novel class of emulsifiers with the aim of generating mucoadhesive emulsions;^21^ the emulsification of medium chain triglycerides was reported with varying stabilities, especially if the thiomers used were able to oxidise or crosslink prior to emulsification. These emulsions were also shown to adhere to porcine buccal mucosa, hence demonstrating the concept of mucoadhesive oil-in-water (O/W) emulsions.

**Fig. 1:**
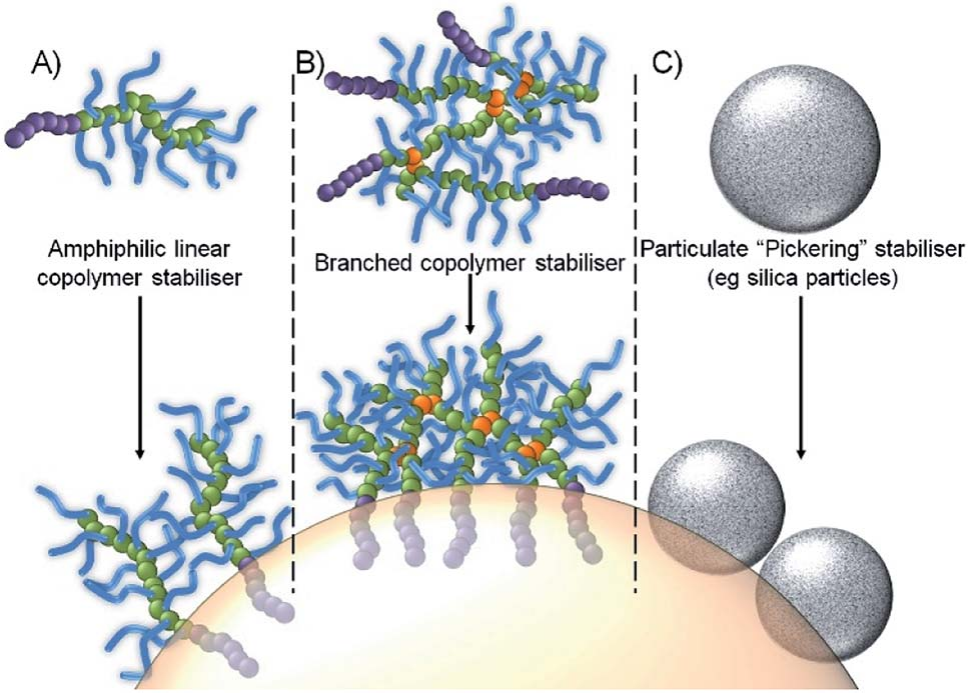
Schematic representation of an oil-in-water emulsion droplet stabilized by: (A) conventional amphiphilic linear copolymer stabilisers; (B) branched copolymer stabiliser with multiple attachment through hydrophobic chain-ends; and (C) particle adsorption.

In recent work, we have shown that branched vinyl polymers made by conventional and controlled radical polymerisation are able to act is highly efficient emulsifiers.^22–25^ Branching was achieved by the copolymerisation of the macromonomer oligo(ethylene glycol methacrylate), (OEGMA) with the divinyl monomer ethylene glycol dimethacrylate (EGDMA), leading to a large number of hydrophilic primary polymer chains bridged by a small number of branch points. Through the utilization of either hydrophobic initiators (controlled radical polymerisation) or hydrophobic chain transfer agents (conventional radical synthesis), amphiphilicity can be introduced into the hydrophilic water-soluble branched polymer at the end of each conjoined primary chain, Fig. 1B. The resulting high molecular weight branched copolymers interact with oil droplet surfaces through multiple attachment sites, providing a contrast to linear polymer surfactants which suffer from single dynamic interactions, and making them more akin to Pickering emulsions, Fig. 1C, as evidenced by their remarkable stability and resistance to dilution.^24^

Herein, we aimed to investigate the impact of decreasing the number of hydrophobic chain-ends within the branched copolymer stabiliser as a strategy for introducing thiol functionality to emulsion droplets and controllably inducing mucoadhesion. Within this model study we monitored emulsion storage stability over prolonged periods and the triggering of demulsification on contact with biosimilar mucus surfaces. To the best of our knowledge, this represents the first demonstration of a triggered mucus-responsive emulsion system enabled by novel branched copolymer emulsifiers.

## Results and discussion

### Investigating chain-end criticality for branched copolymer emulsifiers

#### Polymerisation studies and copolymer characterisation

**Scheme 1:**
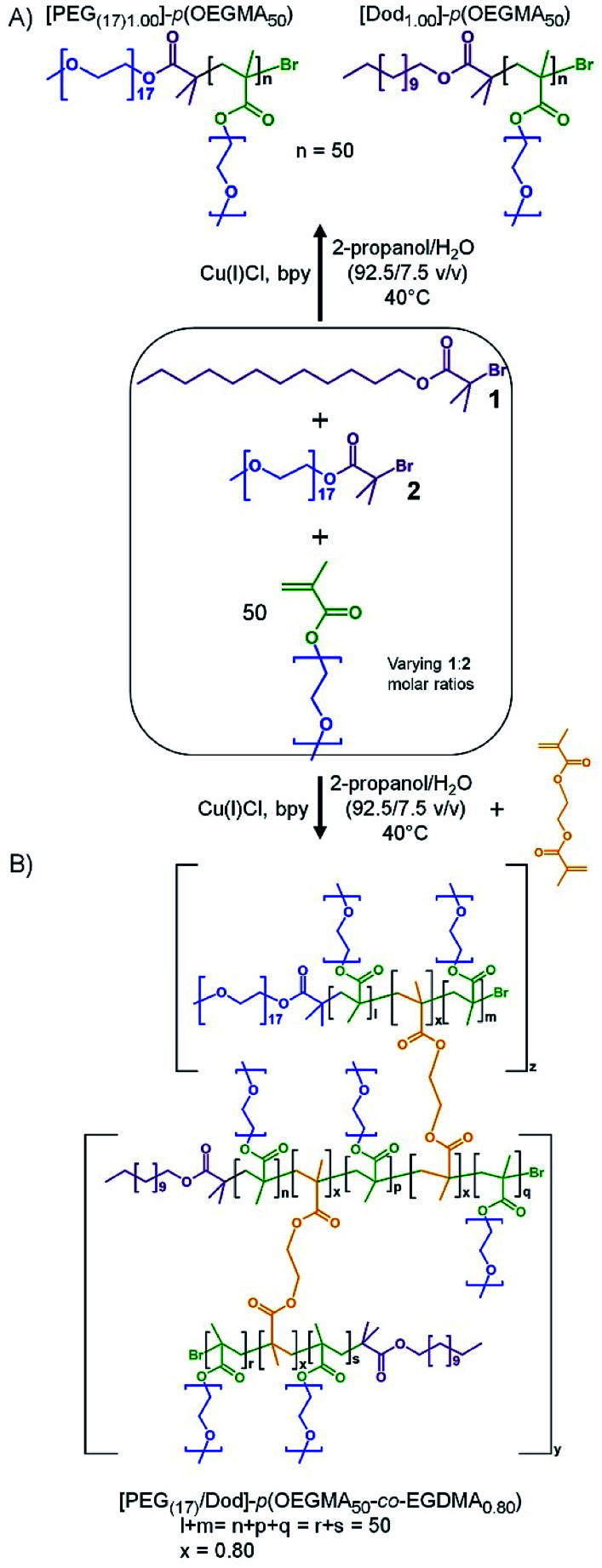
Co-initiation of alcoholic atom transfer radical copolymerisation of oligo(ethylene glycol methacrylate) and ethylene glycol dimethacrylate to form: (A) a mixed linear polymer sample containing hydrophilic [PEG_(17)1.00_]–*p*(OEGMA_50_) and amphiphilic [Dod_1.00_]–*p*(OEGMA_50_) chains, and (B) the branched copolymer with varying ratios of PEG_17_ and dodecyl chain ends.

The synthesis of branched vinyl copolymers using the incorporation of low concentrations of divinyl monomers (less than 1 per propagating primary chain) has been the subject of several research reports.^26^ The mixing of differing monovinyl monomer chemistries within the primary chains of the complex polymer architectures^27,28^ has also been used to introduce main-chain functionality;^22,23^ however, the joining together of polymer chains *via* this approach opens a unique opportunity to also control chain-end functionality, as demonstrated by our group in the formation of hyperbranched-polydendrons with varying combinations of dendritic and polyethylene glycol (PEG) chains.^29–31^ As mentioned above, we have recently reported the formation and application of branched copolymers consisting of OEGMA (*M*_n_ = 300 g mol^−1^) and EGDMA, initiated by dodecyl α-bromoisobutyrate, 1 (Dod-Br, Scheme 1), under atom transfer radical polymerisation (ATRP) conditions, as highly efficient polymeric surfactants; the copolymers contain a hydrophobic dodecyl group at every chain-end.^24^

We hypothesised that initiating this copolymerisation with mixed initiator feedstocks would allow the creation of branched copolymers with varying chain-end composition; however, the decrease in hydrophobic chain-ends may potentially impair the surfactant behaviour of the branched copolymers. To study this in detail, a series of branched copolymers with systematically varying numbers of hydrophilic and hydrophobic chain-ends was synthesised to establish the critical content of hydrophobic (lipophilic) chain-ends within the branched copolymer structures required for stable O/W emulsion formation.

As seen in Scheme 1A, co-initiation of the linear polymerisation of OEGMA with varying ratios of dodecyl-derived, 1, and PEG_17_-derived, 2 (PEG_(17)_-Br; starting material PEG-mono methyl ether 750 g mol^−1^), initiators leads to a mixed linear polymer product where a fraction of the polymer chains possess hydrophilic PEG chain-ends ([PEG_(17)1.00_]–*p*(OEGMA_50_)), and hence are exclusively hydrophilic, whilst the remaining fraction will carry a hydrophobic dodecyl chain-end ([Dod_1.00_]–*p*(OEGMA_50_)), and possess amphiphilic character. Conversely, the introduction of a low molar concentration of EGDMA, Scheme 1B, will combine the mixed linear population into a range of larger structures with an overall controlled average of PEG_17_ and dodecyl chain-ends across the full polymer distribution. If a substantial number of hydrophobic dodecyl chain-ends could be replaced with hydrophilic PEG_17_ chain-ends, whilst maintaining emulsifier properties, an indication of chain-end redundancy would be derived and the potential for utilising the redundancy to introduce functional groups may become available.

A series of branched OEGMA/EGDMA copolymers were, therefore, synthesised *via* a mixed initiator, copper-catalysed ATRP strategy in an isopropyl alcohol (IPA)/water mixture (92.5/7.5 v/v%) at 40 °C. All coinitiated copolymerisations targeted a number average degree of polymerisation (DP_n_) of 50 monomer units ([M]_0_/Σ[I]_0_ = 50) to ensure consistency for the primary chains within the various targeted branched structures. The mixed initiator ratios used systematically varying 1 : 2 molar ratios of 1.00 : 0.00, 0.90 : 0.10, 0.75 : 0.25, 0.50 : 0.50, 0.25 : 0.75, 0.10 : 0.90 and 0.00 : 1.00; a consistent EGDMA : Σ[I]_0_ molar ratio of 0.80 : 1 was also employed ([B]_0_/Σ[I]_0_ = 0.80) as this ratio allows a significant degree of branching whilst avoiding gelation. The linear polymers [PEG_(17)1.00_]–*p*(OEGMA_50_) and [Dod_1.00_]–*p*(OEGMA_50_) were also synthesised, to allow comparison of synthesis conditions for each initiator and emulsification performance of mixed polymer samples as a control. Branched copolymerisations often require extended reaction times, therefore each reaction was monitored by ^1^H nuclear magnetic resonance (NMR) spectroscopy and terminated when high conversion (≥99%) was established. Triple detection size exclusion chromatography (TD-SEC), using a dimethylformamide (DMF)/0.01 M LiBr eluent, was utilised to determine number average molecular weight (*M*_n_), weight average molecular weight (*M*_w_) and dispersity (*Đ*), Table 1.

**Table 1:** Triple detection size exclusion chromatography analysis of OEGMA-based linear homopolymers and branched copolymers initiated using varied molar ratios of Dod-Br and PEG_17_-Br

Architecture	Copolymer	*M*_n_ a (g mol^−1^)	*M*_w_ a (g mol^−1^)	*Đ* a
Linear homopolymers	[Dod_1.00_]–*p*(OEGMA_50_)	30 750	39 750	1.29
	[PEG_(17)1.00_]–*p*(OEGMA_50_)	38 000	70 350	1.85
Branched copolymers	[Dod_1.00_]–*p*(OEGMA_50_-*co*-EGDMA_0.80_)	57 600	235 700	4.09
	[Dod_0.90_/PEG_(17)0.10_]–*p*(OEGMA_50_-*co*-EGDMA_0.80_)	22 300	136 100	6.10
	[Dod_0.75_/PEG_(17)0.25_]–*p*(OEGMA_50_-*co*-EGDMA_0.80_)	62 500	257 700	4.12
	[Dod_0.50_/PEG_(17)0.50_]–*p*(OEGMA_50_-*co*-EGDMA_0.80_)	54 500	163 700	3.00
	[Dod_0.25_/PEG_(17)0.75_]–*p*(OEGMA_50_-*co*-EGDMA_0.80_)	76 400	410 600	5.37
	[Dod_0.10_/PEG_(17)0.90_]–*p*(OEGMA_50_-*co*-EGDMA_0.80_)	40 050	303 800	7.59
	[PEG_(17)1.00_]–*p*(OEGMA_50_-*co*-EGDMA_0.80_)	63 600	169 800	2.66

a TD-SEC using DMF/0.01 M LiBr eluent; all polymerisation achieved ≥99% monomer conversion as determined by ^1^H NMR (CDCl_3_,400 MHz).

The *M*_n_ values of the linear polymer samples were higher than targeted, with *Đ* values indicating non-ideal initiator efficiencies and the inherent dispersity of the PEG_17_-derived macroinitiator. The branched copolymerisations showed relatively consistent *M*_n_ values, in general, suggesting only a minor difference in the behaviour of the two initiators and comparable initiator efficiencies during the early stages of copolymerisation.

**Fig. 2:**
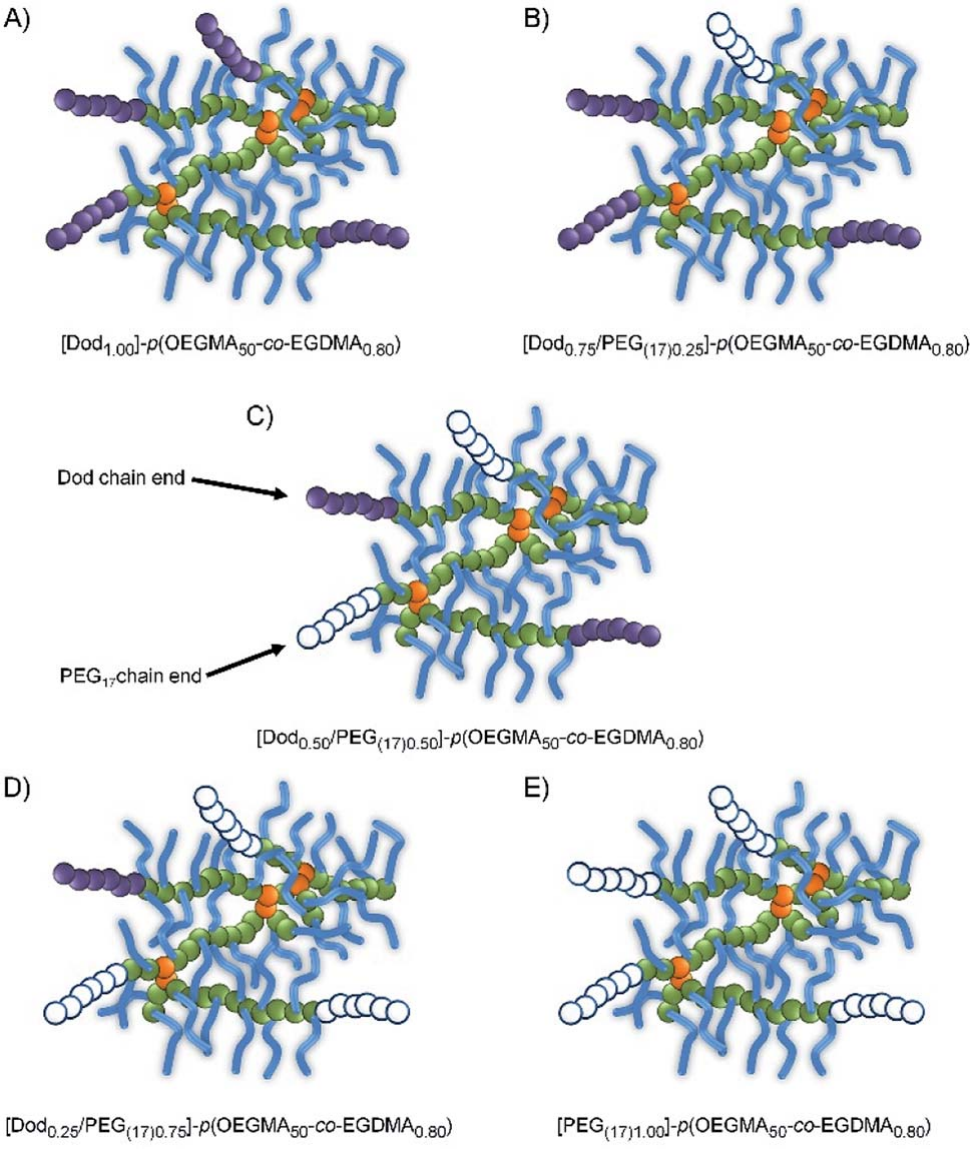
Schematic representation of branched copolymer structures with varying hydrophobic/hydrophilic chain-end compositions.

As expected, the *M*_n_ and *M*_w_ values were consistently higher for the branched copolymers than their linear analogues with correspondingly higher dispersities (ESI Fig. S7–S9†). Within these broad distributions, a weight fraction analysis of the TD-SEC data indicated that between approximately 6–34 wt% of the various samples have molecular weights >500 kg mol^−1^ (therefore containing > 10 conjoined chains), ESI Fig. S10, S11 and Table S1.† As such, although there will be heterogeneity of the mixed initiator-derived chain-ends, a significant proportion of branched copolymers will possess mixtures of PEG and Dod chain-ends even at the extremes of the 1 : 2 ratios targeted, Fig. 2.

### Aqueous solution behaviour and emulsification studies

All branched copolymers showed high aqueous solubility (>5 wt%) and no observable solution differences across the samples with varying overall chain-end composition; lower critical solution temperature (LCST) studies exhibited a trend to lower values with increasing hydrophobic chain-end content, and branched [PEG_1.00_]–*p*(OEGMA_50_-*co*-EGDMA_0.80_) showed slightly lower values than its linear analogue; a relatively narrow range of transition temperatures was observed across the series (approximately 61–67 °C), ESI, Fig S12.†

**Fig. 3:**
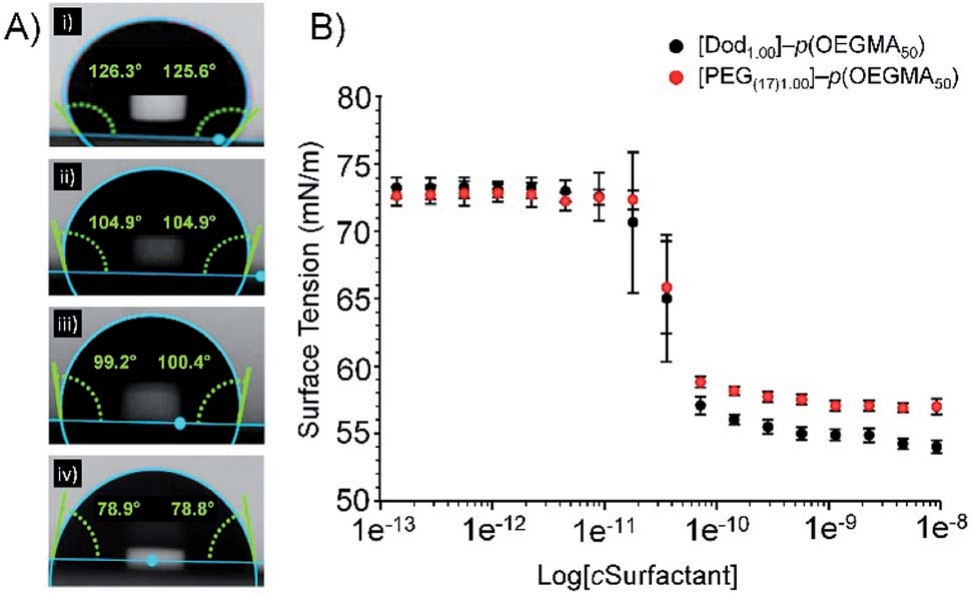
Assessment of branched copolymer aqueous solution behaviours. (A) The relationship between branched copolymer end-group composition and contact angle observed for droplets of aqueous copolymer solutions on a hydrophobic PTFE substrate, where (i) pure water, (ii) [PEG_(17)_]–*p*(OEGMA_50_-*co*-EGDMA_0.80_), (iii) [Dod_0.75_/PEG_(17)0.25_]–*p*(OEGMA_50_-*co*-EGDMA_0.80_) and (iv) [Dod_1.00_]–*p*(OEGMA_50_-*co*-EGDMA_0.80_). (B) Overlaid plots obtained from surface tensiometry measurements of aqueous linear homopolymer solutions of [Dod_1.00_]–*p*(OEGMA_50_) (black circles) and [PEG_(17)1.00_]–*p*(OEGMA_50_) (red circles).

The varying branched copolymer structures, Fig. 2, were also studied using static contact angle measurement of aqueous branched copolymer surfactant solutions (5 wt%). Aqueous solutions were placed onto a hydrophobic polytetrafluoroethylene substrate to evaluate the presence and impact of the varying hydrophilic/hydrophobic chain-ends (Fig. 3A(i)–(iv)). All solutions containing branched copolymers showed a decrease in contact angle from deionised water (125° ± 3.6°, Fig. 3A(i)), indicating the presence of the branched copolymer at the air/water interface and an increase in hydrophobicity (ESI, Table S2†). The lowest contact angle was observed for [Dod_1.00_]–*p*(OEGMA_50_-*co*-EGDMA_0.80_) solutions (80° ± 2.2°) and the introduction of PEG_(17)_-derived chain-ends increased the contact angle of the various solutions to range from approximately 99–105°; the clear inference being that redundant hydrophobic chain-ends are present at the droplet surface for solutions containing [Dod_1.00_]–*p*(OEGMA_50_-*co*-EGDMA_0.80_) and these are somewhat removed and replaced by the more hydrophilic PEG_17_ chain-ends in the solutions of copolymers containing mixed chain-end functionality. The contribution of the hydrophobic polymethacrylate backbone to the changes in observed contact angles must also not be ignored; however, this remains consistent within the polymer structures and may become more prominent with the changing chain-end chemistry.

Surface tension measurements were conducted to study the impact of the increasing number of hydrophilic PEG-derived chain-ends within the branched copolymer structures (ESI, Table S3, Fig. S13†). Interestingly, changes to copolymer end-group functionality across all materials had little impact on their CMC behaviour. The intercept of the first plateau of the surface tension curves were observed at concentrations of between 4.72 × 10^−8^ (±8%) and 7.40 × 10^−8^ (±2%) mg L^−1^ for linear polymers [Dod_1.00_]–*p*(OEGMA_50_) and PEG_(17)1.00_–*p*(OEGMA_50_) respectively (Fig. 3B), suggesting a role for the hydrophobic methacrylate backbone in adsorption to the oil droplet surface. Whilst the slightly lower CMC obtained for [Dod_1.00_]–*p*(OEGMA_50_) could be attributed to the presence of the hydrophobic dodecyl chain-ends, comparable CMC values likely arise due to the low weight fraction of the polymer chains-ends (<2 wt% in all cases based on *M*_n(GPC)_).

This is supported by the consistent CMC values obtained for branched copolymers containing mixed chain-end functionality (ESI Fig. S14†). All branched copolymer surfactants showed complex surface tension behaviour with increasing copolymer concentration; initial plateaus were followed by subsequent further decreases in surface tension at higher concentration. This behaviour is well reported for complex mixtures such as polymeric/small molecule surfactant combinations, and therefore is readily rationalised as being derived from the distribution of species within the statistically branched copolymer molecular weight distribution (ranging from linear polymers through to highly branched structures). CMC values obtained from branched copolymers containing mixed chain-end functionality ranged from 1.08 × 10^−8^ (±16%) to 4.74 × 10^−8^ (±2%) mg L^−1^ and showed no clear trend between chain-end composition and CMC values. This data indicates that, despite their contrasting chain-end compositions, branched copolymer emulsifiers are expected to adopt similar conformations in aqueous solution and would exhibit similar responses to changes in concentration. Furthermore, this demonstrates that it would be possible to manipulate branched copolymer emulsifying properties with minimal impact on their aqueous solution behaviour.

**Fig. 4:**
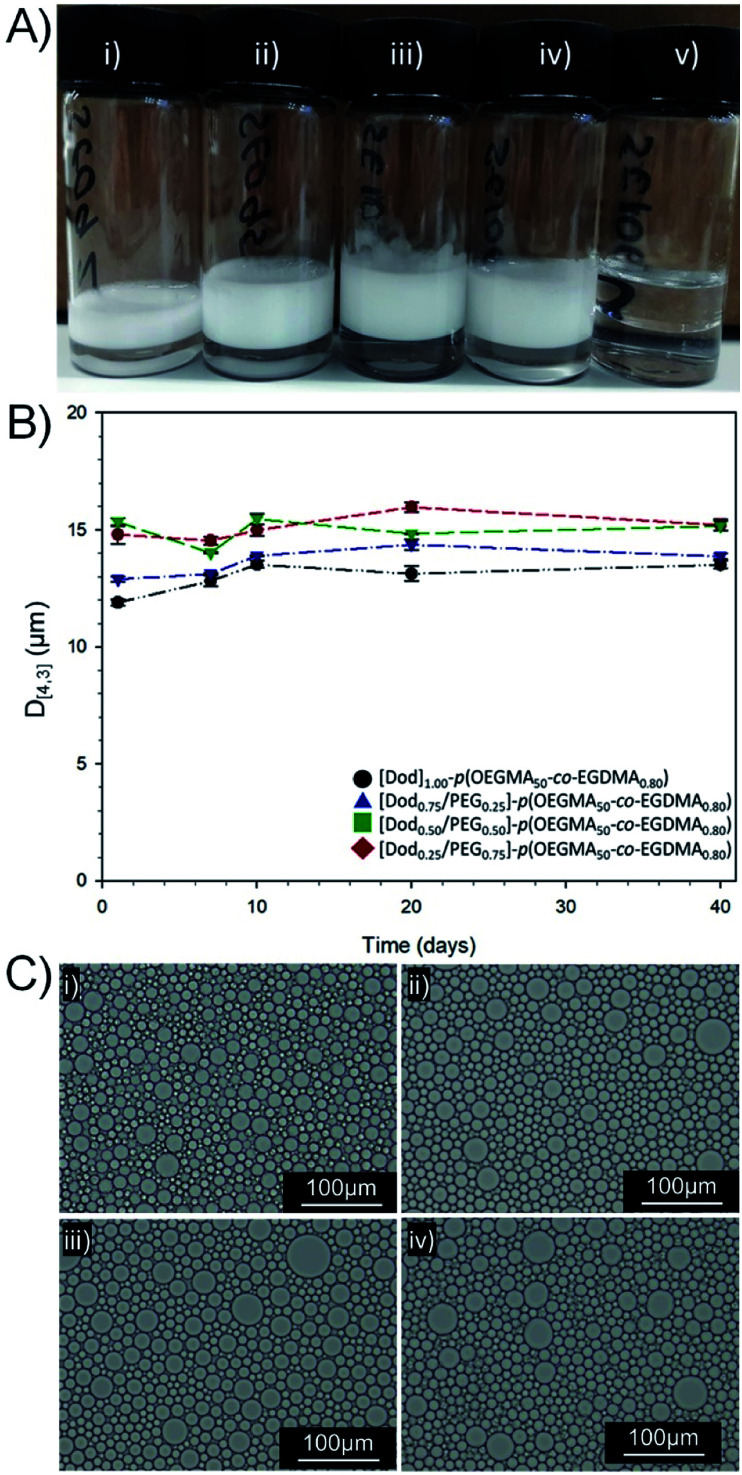
Analysis of O/W emulsions stabilised by branched copolymer emulsifiers with mixed chain-end functionality. (A) Digital photographs taken after 3 years storage at ambient temperature of dodecane-in-water emulsions stabilised with: (i) [Dod_1.00_]–*p*(OEGMA_50_-*co*-EGDMA_0.80_), (ii) [Dod_0.75_/PEG_(17)0.25_]–*p*(OEGMA_50_-*co*-EGDMA_0.80_), (iii) [Dod_0.50_/PEG_(17)0.50_]–*p*(OEGMA_50_-*co*-EGDMA_0.80_), (iv) [Dod_0.25_/PEG_(17)0.75_]–*p*(OEGMA_50_-*co*-EGDMA_0.80_) and (v) [PEG_(17)1.00_]–*p*(OEGMA_50_-*co*-EGDMA_0.80_). (B) Study of emulsion droplet stability over 40 days (laser diffraction) and (C) optical microscopy images of dodecane emulsions after 11 months storage at ambient temperature; (i) [Dod_1.00_]–*p*(OEGMA_50_-*co*-EGDMA_0.80_), (ii) [Dod_0.75_/PEG_(17)0.25_]–*p*(OEGMA_50_-*co*-EGDMA_0.80_), (iii) [Dod_0.50_/PEG_(17)0.50_]–*p*(OEGMA_50_-*co*-EGDMA_0.80_), (iv) [Dod_0.25_/PEG_(17)0.75_]–*p*(OEGMA_50_-*co*-EGDMA_0.80_), 20× magnification, scale bar = 100 μm.

Evaluation of the branched copolymers as emulsifiers (5 mg mL^−1^) was conducted using high shear homogenisation of dodecane (1 : 1 v/v ratio) to create O/W emulsions, Fig. 4. Dodecane was selected as the disperse phase of the O/W emulsions due to its similarity to the dodecyl hydrophobic chain-end, introduced by the Dod-Br initiator, and the favourable interactions that should therefore occur with the resultant oil droplets. In the absence of a branched copolymer emulsifier, stable emulsions could not be produced, with rapid demulsification and resulting phase separation within approximately two minutes of standing; similarly, all samples emulsified with linear polymers, or mixtures of linear polymers with varying hydrophilic/hydrophobic ratios of chain ends, demulsified relatively rapidly on standing for less than one week (ESI Fig. S15†).

For all samples containing branched copolymer surfactants with various ratios of Dod and PEG_(17)_ chain-ends, stable emulsions were formed. Creaming of the O/W emulsion was observed after 24 hours equilibration due to density differences; however, no oil separation was apparent with emulsions characteristically white and opaque and stable for >3 years at ambient temperature (Fig. 4A(i)–(iv)). Quantitative analysis by laser diffraction spectroscopy (ESI, Table S4, Fig. S16†) showed highly comparable volume average mean diameters (*D*_[4,3]_) ranging from 11.9–15.3 μm with a trend towards larger droplet diameters with decreasing Dod content within the branched copolymer solutions of identical concentration. Interestingly, the size distribution of O/W emulsion droplets stabilised by branched copolymer emulsifiers resembled the smaller droplets within the bimodal size distribution generated by the equivalent mixed linear homopolymer system, described above (ESI Fig. S17†).

The reduction of the number of hydrophobic chain-ends appears to be analogous to a reduction in small-molecule surfactant concentration, therefore a lower surface area may be stabilised and a smaller number of larger droplets result. As mentioned above, the dispersity of the co-initiated branched copolymer samples does also lead to the potential presence of a fraction of linear [PEG_(17)1.00_]–*p*(OEGMA_50_) and some lightly branched materials having no Dod chain-ends. As may have been predicted, emulsions prepared using [PEG_(17)1.00_]–*p*(OEGMA_50_-*co*-EGDMA_0.80_) showed no long-term stability, with complete and rapid demulsification occurring within 72 hours (ESI Fig. S18†). The role of the hydrophobic backbone in generating some stability to the emulsions cannot be ruled out; however, this material emphasises the critical importance of chain-end chemistry on the formation of stable emulsions.

**Fig. 5:**
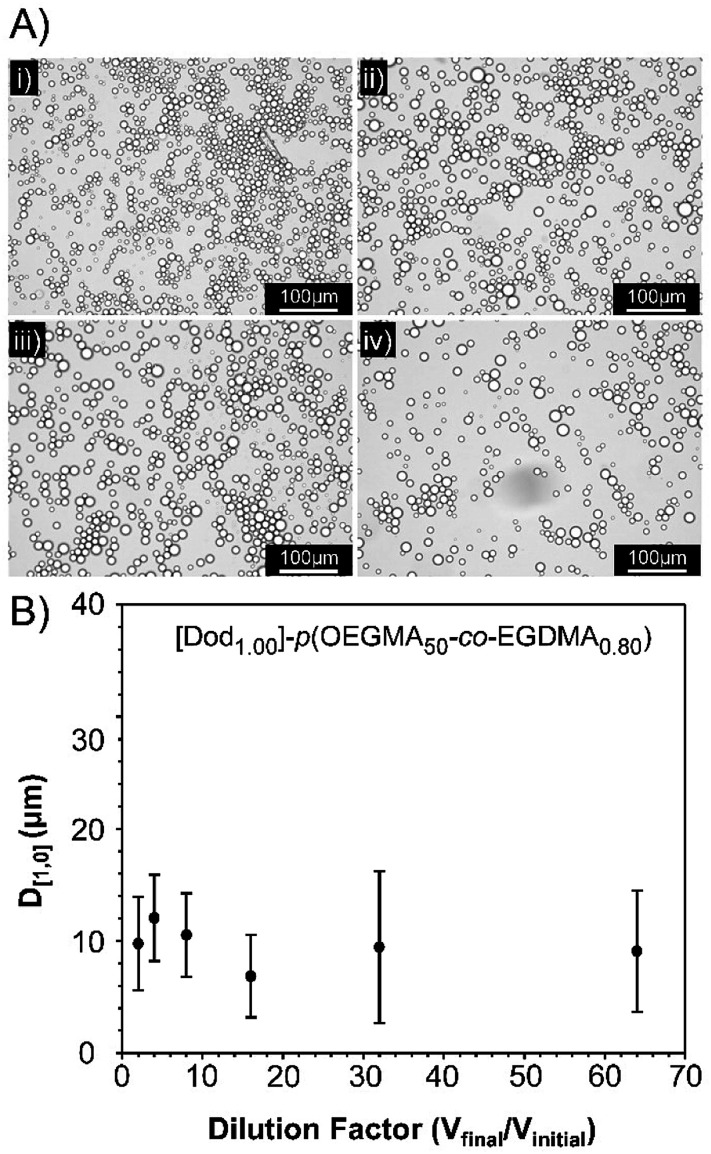
Dilution stability of a [Dod_1.00_]–*p*(OEGMA_50_-*co*-EGDMA_0.80_)-stabilised dodecane-in-water emulsion. (A) Optical microscopy images at dilution factors of; (i) 2-fold, (ii) 4-fold, (iii) 6-fold, and (iv) 32-fold. (B) Measurement of droplet diameters following dilution in deionised H_2_O, as determined by optical microscopy (±std. deviation).

Observation of the *D*_[4,3]_ values over 40 days under ambient conditions for the emulsions stabilised with Dod-containing branched copolymers revealed very small changes in emulsion droplet sizes overall (Fig. 4B). The obtained *D*_[4,3]_ values correlated well with those obtained by optical microscopy imaging (Fig. 4C(i)–(iv)), which show well-defined spherical droplets with no signs of coalescence across varying branched copolymer compositions, even after several months of storage under ambient conditions. Laser diffraction was also used to assess long-term stability of the different O/W emulsions and minimal changes in the observed distributions and measured *D*_[4,3]_ values were seen between samples studied 24 hours after emulsification and after storage under ambient conditions for 3 years (ESI, Fig. S19, Table S5†). Dilution of the Dod-containing branched copolymer stabilised emulsions from the high concentration creamed layer through to 6.25% (v/v) also confirmed the unusual stability of the emulsions (Fig. 5) as reported previously for branched copolymer-stabilised O/W nanoemulsions.^24^ This behaviour is remarkable given the probable presence of linear and low molecular weight species within the distribution bearing no hydrophobic chain-ends.

### Thiol functional to branched copolymer emulsifiers

**Scheme 2:**
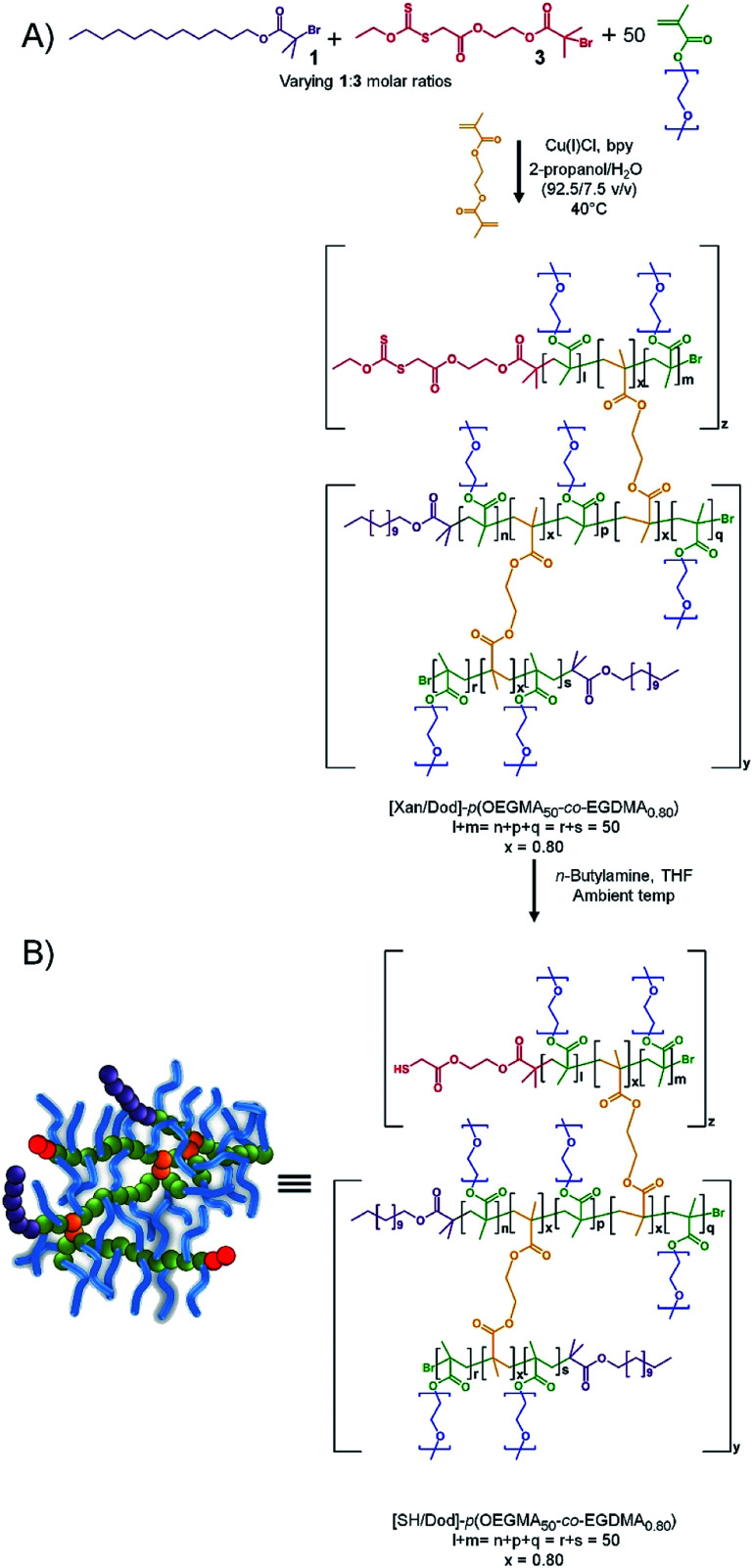
Preparation of thiol functional branched copolymer emulsifiers. (a) Incorporation of protected thiols (xanthates) at polymer end-groups *via* co-initiated ATRP of OEGMA in the presence of EGDMA. (b) Thiol deprotection *via* aminolysis to expose thiol functionalities at pre-determined end-group compositions.

The ability to introduce redundant chain-ends into the branched copolymer emulsifiers, whilst maintaining efficient emulsification, offers the potential to add functional groups to the termini of a controlled number of primary chains across the polymer distribution without compromising performance of the emulsifiers. Here, we have targeted thiol functionality, to facilitate mucus adhesion, through the use of a mixed initiation using 1 and a previously reported xanthate-functional ATRP initiator (Xan-Br), 3, Scheme 2.

As previously reported for analogous materials, the resulting [Xan_*y*_/Dod_*x*_]–*p*(OEGMA_50_-*co*-EGDMA_0.80_) branched copolymers, with varying ratios of Dod and xanthate chain ends, may be readily deprotected using *n*-butylamine to liberate the thiol functionality and create a series of functional emulsifiers denoted as [SH_*y*_/Dod_*x*_]–*p*(OEGMA_50_-*co*-EGDMA_0.80_). The formation of linear polymers under identical conditions to those used above but initiated with 3, to yield [Xan_1.00_]–*p*(OEGMA_50_), was investigated and shown to be comparable to the synthesis of [Dod_1.00_]–*p*(OEGMA_50_) and [PEG_(17)1.00_]–*p*(OEGMA_50_). The initiator efficiency of 3 does appear to be slightly higher than the other initiators (approximately 67% for 3; approximately 50% for 1) suggesting a slightly higher number of actual xanthate-bearing chains would be present in branched copolymer samples using the initiator than targeted through the nominal initiator molar ratios. The formation of a series of comparable branched copolymerisations utilising EGDMA and mixed 1 : 3 coinitiation achieved ≥99% monomer conversion in all cases and samples were analysed by TD-SEC to determine molecular weights and dispersities, Table 2 (ESI, Fig. S20 and S21†).

**Table 2:** Triple detection size exclusion chromatography analysis of xanthate-containing (co)polymers

Polymer	*M*_n_ a (g mol^−1^)	*M*_w_ a (g mol^−1^)	*Đ* a
Xan_1.00_–*p*(OEGMA_50_)	22 800	39 300	1.72
[Xan_0.08_/Dod_0.92_]–*p*(OEGMA_50_-*co*-EGDMA_0.80_)	45 850	297 050	6.48
[Xan_0.25_/Dod_0.75_]–*p*(OEGMA_50_-*co*-EGDMA_0.80_)	38 250	147 500	3.86
[Xan_0.50_/Dod_0.50_]–*p*(OEGMA_50_-*co*-EGDMA_0.80_)	52 000	401 500	7.72
[Xan_0.75_/Dod_0.25_]–*p*(OEGMA_50_-*co*-EGDMA_0.80_)	70 750	281 900	3.98
[Xan_0.90_/Dod_0.10_]–*p*(OEGMA_50_-*co*-EGDMA_0.80_)	37 150	134 500	3.62
[Xan_0.95_/Dod_0.05_]–*p*(OEGMA_50_-*co*-EGDMA_0.80_)	64 800	367 200	5.67

a TD-SEC using DMF/0.01 M LiBr eluent (60 °C); all polymerisations achieved ≥99% monomer conversion.

To compare the xanthate-functional branched copolymers with those containing mixed Dod/PEG_17_ chain-ends, the TD-SEC data was analysed to provide a weight fraction analysis, indicating that, again, between approximately 6–19 wt% of the species in branched copolymer samples have molecular weights >500 kg mol^−1^ which equates to >10 conjoined primary chains (ESI, Fig. S22 and Table S6†). This was highly encouraging as the weight fractions of high molecular weight, highly branched structures are broadly similar to those shown to act as efficient emulsifiers. Removal of the xanthate protecting group, and liberation of the thiol-functionality, was conducted for a selected series of functional copolymers, leading to the systematically-varying thiol-functional copolymers [SH_0.25_/Dod_0.75_]–*p*(OEGMA_50_-*co*-EGDMA_0.80_), [SH_0.50_/Dod_0.50_]–*p*(OEGMA_50_-*co*-EGDMA_0.80_), [SH_0.75_/Dod_0.25_]–*p*(OEGMA_50_-*co*-EGDMA_0.80_) and [SH_0.95_/Dod_0.05_]–*p*(OEGMA_50_-*co*-EGDMA_0.80_). ^1^H NMR analysis was used to confirm the removal of xanthate groups from branched copolymer chain-ends post deprotection evident by the disappearance of the chemical shift attributed to the methylene protons of the xanthate group at 4.66 ppm (ESI, Fig. S23†).

Aqueous solutions of these materials were studied using surface tensiometry, and similar complex behaviour was observed (ESI, Fig. S24†), with the first plateau regions observed at 9.12 × 10^−6^ (±8.7%), 9.13 × 10^−6^ (±8.4%), 1.55 × 10^−5^ (±16.2%) and 1.83 × 10^−5^ (±2.0%) mg mL^−1^ respectively (ESI, Table S7†). This correlation of surface tension with thiol content was more marked than the previous Dod/PEG materials and the lack of the additional PEG chain-ends generated values more consistent with those seen when studying [Dod_1.00_]–*p*(OEGMA_50_-*co*-EGDMA_0.80_); this is probably due to the hydrophobic nature of the xanthate-bearing chain-ends. Contact angle measurements were also within the same range as those seen for Dod/PEG branched copolymers containing varied chain-end compositions (93–108°, ESI, Fig. S25 and Table S8†) and efficient emulsification was, therefore, expected.

### Emulsification and mucus-responsive studies using thiol-functional to branched copolymer stabilisers

The functional-emulsions initially envisaged for these studies require biologically/pharmacologically-relevant oil phases to be stabilised in water. As such, and building on our recent reports,^24,31^ emulsions were generated using the naturally occurring polyunsaturated liquid hydrocarbon squalene using the thiol-functional branched copolymers. This more detailed study was limited to [SH_0.75_/Dod_0.25_]–*p*(OEGMA_50_-*co*-EGDMA_0.80_) due to its balance of high thiol content, a predicted efficient number of Dod chain-ends to maintain emulsification performance, a good balance of molecular weight, and a weight fraction of highly branched chains representing a high probability of thiol content (8 wt% ≥ 19 chains; 4 wt% ≥ 28 chains; and 2 wt% ≥ 37 conjoined chains) (ESI, Table S6†).

**Fig. 6:**
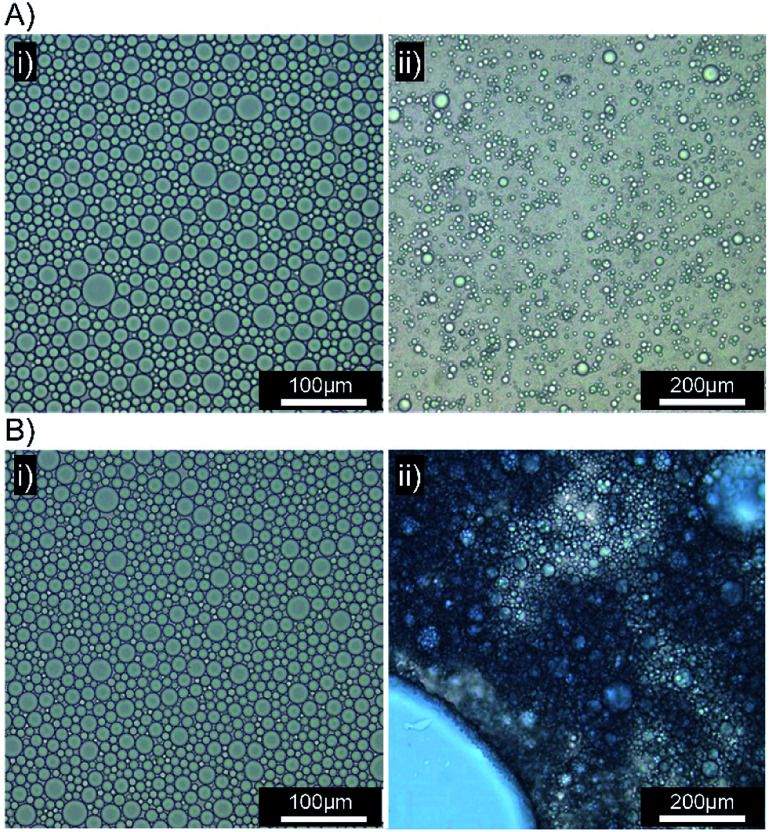
Optical microscopy images of squalene-in-water emulsions containing Oil Blue A (0.1 wt%). (A) [Dod_1.00_]–*p*(OEGMA_50_-*co*-EGDMA_0.80_)- and (B) [SH_0.75_/Dod_0.25_]–*p*(OEGMA_50_-*co*-EGDMA_0.80_)-stabilised emulsions both (i) before and (ii) 10 minutes after addition to a biosimilar mucosal surface.

**Fig. 7:**
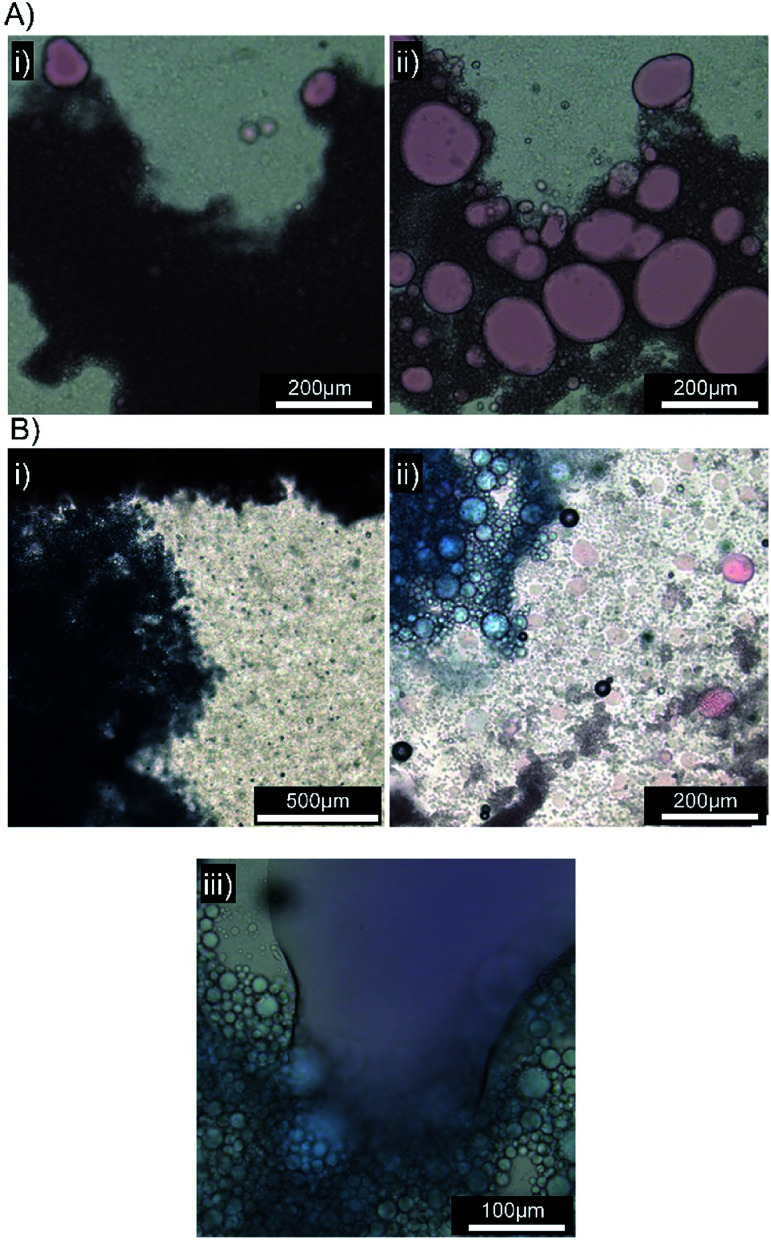
Optical microscopy images of emulsions in the presence of a biosimilar mucosal surface. (A) Emulsion stabilised by [SH_0.75_/Dod_0.25_]–*p*(OEGMA_50_-*co*-EGDMA_0.80_) with Oil Red O (0.1 wt%), (i) immediately after application, (ii) after 10 minutes. (B) Emulsions stabilised by [SH_0.75_/Dod_0.25_]–*p*(OEGMA_50_-*co*-EGDMA_0.80_) with Oil Red O (top) and Oil Blue A (bottom) (i) immediately after application, (ii) after 10 minutes and (iii) presence of a mixed mobile liquid oil layer.

Squalene emulsions were generated under identical conditions to dodecane emulsions described above and, as expected, this [SH_0.75_/Dod_0.25_]–*p*(OEGMA_50_-*co*-EGDMA_0.80_) branched copolymer acted as a highly efficient emulsifier, generating approximately 16 μm oil droplets (*D*_[4,3]_) within an aqueous continuous phase (ESI, Table S9†). The potential for disulfide bond formation on storage, leading to possible aggregation or demulsification, was studied by storing the thiol-functional emulsion under atmospheric conditions and ambient temperature for extended periods. Laser diffraction measurements taken each week for 4 weeks showed no meaningful variation or effect of storage and no visible separation of oil was observed (ESI Fig. S26†). Squalene is a good solvent for a range of molecules including two dye molecules, the diazo dye Oil Red O and an anthraquinone Oil Blue A, which were studied as models of future therapeutic agents that may be incorporated into the emulsions (Fig. 6 & 7). This was achieved *via* addition of small amounts (0.1 wt% w.r.t. oil phase) of the respective dye into the emulsification process. Incorporation of the dye molecules resulted in slightly smaller emulsions (*D*_[4,3]_ approximately 13 μm) and samples stabilised with either Dod_1.00_–*p*(OEGMA_50_-*co*-EGDMA_0.80_) or [SH_0.75_/Dod_0.25_]–*p*(OEGMA_50_-*co*-EGDMA_0.80_) were generated to investigate the impact of thiol-functionality on the stable emulsions (ESI, Fig. S27†).

Several biosimilar mucus formulations have been reported; a mimic of natural mucus, outlined by Boegh *et al.*,^32^ has been reported as being rheologically optimised to strongly resemble natural porcine intestinal mucus. The biosimilar mucus contains Carbopol 940 (a high molecular weight crosslinked poly(acrylic acid)), to provide a shear-thinning dominant elastic behaviour, whilst chemical similarity is obtained by incorporation of porcine mucin, bovine serum albumin and a lipid mixture of cholesterol, phosphatidylcholine and linoleic acid; the surfactant polysorbate 80 is also present. The biosimilar mucus was spread evenly across a glass microscope slide to provide a mucosal surface for investigation by optical microscopy; each sample was viewed and monitored closely for 10 minutes after the addition of the concentrated emulsions to the mucus substrate. Squalene emulsions stabilised with [Dod_1.00_]–*p*(OEGMA_50_-*co*-EGDMA_0.80_) (Fig. 6A(i)) were unaffected by the mucus surface, spreading evenly across the substrate and maintaining their ability to move throughout the study; ready identification of individual stable droplets was achieved *via* optical microscopy, with no observable aggregation or perturbation of the dispersed oil phase (Fig. 6A(ii)). This behaviour was in marked contrast to the emulsions stabilised with [SH_0.75_/Dod_0.25_]–*p*(OEGMA_50_-*co*-EGDMA_0.80_) (Fig. 6B); addition of this emulsion to the biosimilar mucus-covered glass slide led to instantly observable differences in spreading and aggregation. During the same 10 minute observation period, the formation of free oil after droplet rupturing was also seen (Fig. 6B(ii)).

A separate [SH_0.75_/Dod_0.25_]–*p*(OEGMA_50_-*co*-EGDMA_0.80_)-stabilised squalene-in-water emulsion was prepared, containing Oil Red O (ESI Fig. S28†). Exchange of Oil Blue A for Oil Red O had no impact on the mucus-responsive behaviour, with extensive demulsification occurring within 10 minutes of addition to a biosimilar mucosal surface (Fig. 7A(i) and (ii)).

The mucus-triggered demulsification was investigated further *via* simultaneous addition of the two dye-loaded, thiol-functional emulsions to the same mucus substrate. As seen in previous experiments, the emulsions adhered to the mucus at the point of addition with a clear boundary between the coloured emulsions and limited mixing (Fig. 7B(i)). The resulting rupturing of the oil phases with each zone of coloured emulsion (Fig. 7B(ii)), led to a mobile liquid oil layer that quickly mixed (Fig. 7B(iii)), generating a purple colour, in the presence of distinct and static red and blue areas. This suggests opportunities for the isolation of incompatible oil-soluble chemistries, or combination products, with triggered demuslification and mixing on target mucosal surfaces.

**Fig. 8:**
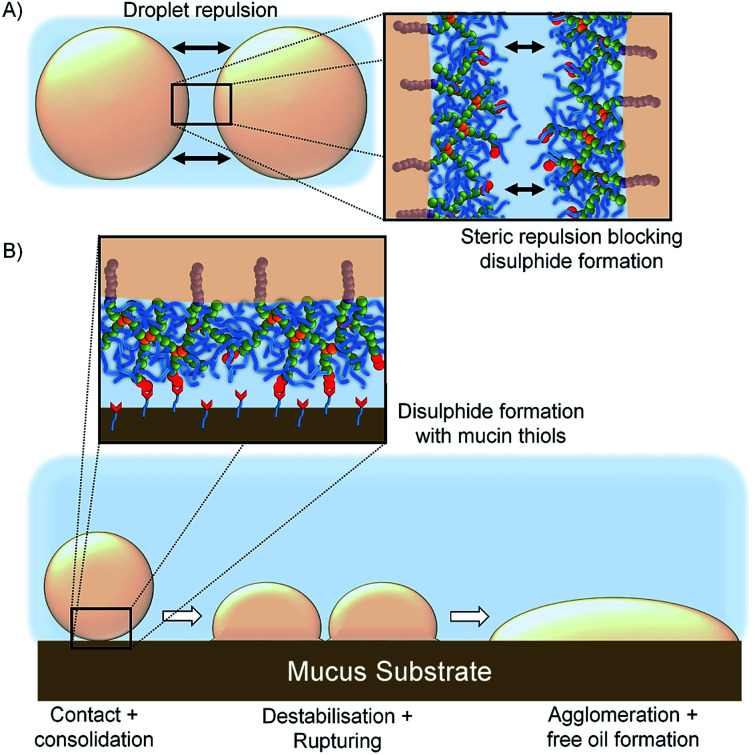
Schematic representation of; (A) repulsion between neighbouring emulsion oil droplets due to the steric hindrance of OEGMA pendant chains leading to high levels of stability and (B) disulfide bond formation between the thiol groups present on the surface of the oil droplets and the mucosal surface leading to droplet destabilisation and release of free oil.

The limited spreading, and observed lack of motion, of the [SH_0.75_/Dod_0.25_]–*p*(OEGMA_50_-*co*-EGDMA_0.80_)-stabilised emulsion suggests rapid and strong mucus interactions, presumably *via* disulfide bond formation between the cysteine-rich regions within the porcine mucin and the thiol functionality presented at the oil droplet surface. Chemical bond formation during the consolidation stage is characteristic of the so-called “second generation” mucoadhesive materials and disulfide bond formation is the basis of most thiolated mucoadhesive macromolecules. This correlates well with the literature “contact/consolidation” model of mucoadhesion but is surprising given the high stability of the thiol-functional droplets in the absence of mucus. We hypothesise that the thiols at the branched copolymer chain ends are unable to approach each other to form bridging disulfide bonds upon storage, due to the steric repulsion of the branched *p*(OEGMA) chains at the oil-droplet/water interface (Fig. 8A); intra-droplet reaction is also highly unlikely given the density of OEGMA side chains along the *p*(OEGMA) backbone. The rapid interaction with the mucus substrate appears to suggest that steric repulsion is not a major factor at this interface, and the thiols present in the cysteine-rich areas of the porcine mucus are able to bond readily with thiol-functional emulsion droplets (Fig. 8B).

The demulsification behaviour, and release of free oil, suggests that the formation of covalent bonds with the mucus substrate leads to either a removal of branched copolymer stabiliser from the oil/water interface, with subsequent destabilisation and agglomeration, or the modification of the contact angle at the mucus–emulsion interface and causing the droplet to rupture and spread resulting in agglomeration and release of the emulsified oil.

## Conclusions

Mucoadhesive materials offer a considerable opportunity for the formulation and delivery of a range of compounds to target mucosal sites. Mucoadhesive emulsions are of particular interest as the ability to dissolve hydrophobic materials within the stabilised oil-droplets offers the potential to carry active molecules and deliver them to specific locations; antimicrobials, antivirals, hygiene agents and drug compounds are of clear interest but materials able to diagnose, label or indicate local environmental conditions also have applications. Here, we have shown a novel strategy for generating bespoke thiol-functional, highly efficient emulsifiers and, to the best of our knowledge, such materials have not been reported previously. The branched copolymer synthesis platform, copolymerising a low concentration of bifunctional monomer within a conventional ATRP polymerisation, allows not only the formation of high molecular weight materials, but also the introduction of amphiphilicity with controlled chain-end functionalisation; the demonstration of chain-end redundancy and thiol-functionality introduction offers new avenues for functional-emulsion research. Mucus-triggered oil release was not an initial target of this study but the ability to form such stable emulsions with rapid release is remarkable and requires additional research to understand both the limitations of the approach and its full opportunities. For simplicity, one branched copolymer emulsifier and one ratio of emulsifier to oil, at one oil-droplet size, were selected for these initial mucus–substrate interaction studies, however, there are options to tailor the interactions through concentration of thiol-functional groups and oil-droplet size; these studies are ongoing.

## Materials and methods

### Materials

Calcium chloride (CaCl_2_), magnesium sulfate (MgSO_4_), sodium hydrogen carbonate (NaHCO_3_), sodium chloride (NaCl), triethylamine (TEA) and hydrochloric acid (HCl, 1 M) were purchased from Fisher Scientific and used as received. Ethylene glycol (99.8%), anhydrous tetrahydrofuran (THF, 99%), 1-dodecanol (99%), α-bromoisobutyl bromide (98%), anisole (99%), oligo(ethylene glycol methacrylate) (OEGMA, *M*_n_ = 300 g mol^−1^, 98%), ethylene glycol dimethacylate (EGDMA), 2,2′-bipyridyl (bpy), copper(i) chloride (CuCl, 97%), activated neutral alumina, basic alumina, porcine mucin, bovine serum albumin (BSA, >98%), cholesterol, linoleic acid, phosphatidylcholine and polysorbate 80 were purchased from Sigma Aldrich and used as received. Potassium ethyl xanthogenate (96%), 2-bromoacetic acid, 4-(dimethylamino) pyridine (DMAP, >99%), *N*,*N*′-dicyclohexylcarbodiimide (DCC, >99%) and 2-bromoacetic acid were all purchased from Alfa Aesar and used as received. 4-(Dimethylamino)pyridinium-4-toluene sulfonate (DPTS) was synthesised as previously reported.^25^ Polyacrylic acid (Carbopol 940) was purchased from Lubrizol. All solvents, unless stated otherwise, were reagent grade, purchased from Fisher Chemicals and used as received.

### Characterisation

Nuclear magnetic resonance (NMR) spectroscopy was recorded using a Bruker DPX-400 spectrometer operating at 400 MHz for ^1^H and 100 MHz ^13^C nuclei respectively. Molecular weight data was obtained by triple detection size exclusion chromatography (TD-SEC) using a Malvern Viscotek SEC_Max_. The instrument was equipped with a GPC_max_ VE2001 auto sampler, two Viscotek D6000 columns, a guard column and a triple detector array TDA305 (refractive index, light scattering and viscometer). TD-SEC was conducted at 60 °C using a mobile phase of DMF containing 0.01 M lithium bromide (LiBr) at a flow rate of 1 mL min^−1^. Emulsifications were conducted using an IKA T25 ULTRA-TURRAX high-shear homogeniser. Oil-in-water (O/W) emulsions were characterised by laser diffraction using a Malvern Mastersizer 2000A equipped with a Hydro 2000 SM dispersion unit. Microscope images were obtained using a Leica DM4 B microscope fitted with a CMOS camera. A Kibron Delta-8 surface tensiometer was used to measure the surface tension of aqueous polymer solutions at 20 °C. The contact angles of aqueous polymer solution droplets rested on a Teflon substrate were measured using A Kruss DSA100E drop shape analyser equipped with an advanced digital camera (×20 magnification).

## Experimental procedures

### Initiator synthesis – 2-dodecyl 2-bromoisobutyrate (Dod-Br); 1

1-Dodecanol (9.32 g, 50.0 mmol, 1.00 equiv.) and TEA (6.07 g, 60.0 mmol, 1.20 equiv.) were dissolved in dichloromethane (DCM, 70 mL). A solution of α-bromoisobutyl bromide (13.8 g, 60.0 mmol, 1.20 equiv.) in DCM was added dropwise to the mixture *via* a pressure equalising dropping funnel and stirred in an ice bath under a nitrogen (N_2_) atmosphere. After addition, the reaction vessel was left to warm to ambient temperature and was left stirring for 24 hours. The solution was washed with NaHCO_3_ (1 M, 1 × 50 mL) and deionised water (4 × 50 mL). The organic layer was then dried over anhydrous MgSO_4_, filtered, and concentrated *in vacuo*. The product was then passed through a basic alumina column. The product, 2-dodecyl 2-bromoisobutyrate (Dod-Br); 1, was isolated as a clear oil in 53% yield (8.91 g) and the correct structure was confirmed using ^1^H and ^13^C NMR spectroscopy (ESI Fig. S1†). ^1^H NMR (400 MHz, CDCl_3_) *δ* (ppm) = 0.81 (t, *J* = 6.6 Hz, 3H), 1.19 (m, 18H), 1.61 (m, 2H), 1.86 (s, 6H, CH_3_), 4.08 (t, *J* = 6.6 Hz, 2H). ^13^C NMR (100 MHz, CDCl_3_) *δ* (ppm) = 14.1, 22.7, 25.8, 28.4, 29–30, 32.0, 56.0, 66.2, 171.8. Mass spectrometry: [MH]^+^ (C_16_H_32_BrO_2_) *m*/*z* = 335.0. Experimentally: ES MS [MH]^+^*m*/z = 335.2. Elemental analysis: calculated (%): (C_16_H_31_O_2_Br) = C, 57.31; H, 9.32. Experimental (%) = C, 57.33; H, 9.20.

### Initiator synthesis – 2-poly(ethylene glycol)_17_ 2-bromoisobutyrate (PEG_17_-Br); 2

Monomethoxy poly(ethylene glycol) (*M*_n_ = 750 g mol^−1^, 23.0 g, 30.7 mmol, 1.00 equiv.) was dissolved in anhydrous THF (40 °C) under a dry N_2_ atmosphere. DMAP (0.038 g, 0.30 mmol, 0.01 equiv.) and TEA (5.60 g, 53.7 mmol, 1.75 equiv.) were added and the reaction mixture stirred in an ice bath. α-Bromoisobutyrl bromide (10.60 g, 46.0 mmol, 1.51 equiv.) was added dropwise using a dropping funnel over 30 minutes and a white precipitate (Et_3_NH^+^Br^−^ salt) was immediately formed. The reaction was left for 24 hours, the precipitate removed by filtration and the solvent removed *in vacuo*; the resulting crude product was precipitated twice from acetone into petroleum ether (30–40 °C). The product, 2-(polyethylene glycol)_17_ 2-bromoisobutyrate (PEG_17_-Br); 2, was isolated as a white wax in 65% yield (15.0 g) and the correct structure was confirmed using ^1^H and ^13^C NMR spectroscopy (ESI Fig. S2†). ^1^H NMR (400 MHz, D_2_O) *δ* ppm = 1.86 (s, 6H), 3.30 (S, 3H), 3.64 (m, 60H), 3.77 (m, 2H), 4.32 (m, 2H). ^13^C NMR (100 MHz, D_2_O) *δ* ppm = 29.8, 56.8, 65.4, 38.3, 69.5, 69.5, 69.7, 70.9, 173.7. Elemental analysis: calculated (%): (C_37_H_73_O_18_Br) = C, 50.16; H, 8.24. Experimental (%) = C, 49.25; H, 8.31.

### Initiator synthesis – 2-(2-((ethoxycarbonothioyl)thio)acetoxy)ethyl 2-bromoisobutyrate (Xan-Br), 3

The synthesis of 2-(2-((ethoxycarbonothioyl)thio)acetoxy)ethyl 2-bromoisobutyrate (Xan-Br); 3, required the preparation of two precursor molecules; 2-((ethoxycarbanothioyl)thio) acetic acid and 2-hydroxyethyl 2-bromoisobutyrate. For the synthesis of 2-((ethoxycarbanothioyl)thio) acetic acid, potassium ethyl xanthogenate (53.1 g, 311.0 mmol, 1.0 equiv.) was added to a two-neck round bottom flask and stirred in acetone (400 mL). A solution of 2-bromoacetic acid (38.4 g, 276.0 mmol, 0.08 equiv.) in acetone (100 mL) was added dropwise to the reaction vessel over 20 minutes using a dropping funnel and the reaction was left to stir at ambient temperature for 16 hours. The crude mixture was filtered under vacuum, the residue washed with acetone and the solvent removed *in vacuo*. The residual oil was solubilised in a minimum amount of dichloromethane and washed with brine (150 mL). The organic layer was dried over MgSO_4_ and the solvent was removed *in vacuo* to give a white solid. The product, 2-((ethoxycarbanothioyl)thio) acetic acid, was isolated in 33% yield (16.28 g) and the correct structure was confirmed using ^1^H and ^13^C NMR spectroscopy (ESI Fig. S3†). ^1^H NMR (400 MHz, CDCl_3_) *δ* ppm = 1.43 (t, *J* = 7.1 Hz, 3H), 3.98 (s, 2H), 4.65 (q, *J* = 7.1 Hz, 2H), 9.3 (s, br, –OH). ^13^C NMR (100 MHz, CDCl_3_) *δ* ppm = 13.70, 37.63, 70.93, 173.86, 212.07. Mass spectrometry: calculated: [MH]^+^ (C_6_H_12_BrO_3_) *m*/*z* = 211.0. Experimental: CI MS [MNa]^+^*m*/z = 211.0. Elemental analysis: calculated (%): (C_5_H_8_O_3_S_2_) = C, 33.32; H, 4.47; S, 35.58. Experimental (%) = C, 32.80; H, 4.40; S, 34.59.

For the synthesis of 2-hydroxyethyl 2-bromoisobutyrate, ethylene glycol (301 g, 4.86 mol, 50.0 equiv.) and TEA (20.3 g, 0.20 mol, 2.00 equiv.) were dissolved in anhydrous tetrahydrofuran (100 mL) and the reaction was stirred in an ice bath. α-Bromoisobutyl bromide (22.32 g, 97.1 mmol, 1.00 equiv.) was added dropwise over 30 minutes and the reaction was left stirring under nitrogen atmosphere at ambient temperature for 16 hours. The reaction mixture was poured into deionised water (800 mL) and extracted with dichloromethane (6 × 100 mL), the retrieved layers were washed with 1 M HCl (2 × 300 mL), dried over MgSO_4_ and the solvent was removed *in vacuo*. The product, 2-hydroxyethyl 2-bromoisobutyrate, was isolated as a clear oil in 91% yield (18.6 g) and the correct structure was confirmed using ^1^H and ^13^C NMR spectroscopy (ESI Fig. S4†). ^1^H NMR (400 MHz, CDCl_3_) *δ* ppm = 1.96 (s, 6H), 3.87 (t, *J* = 4.7 Hz, 2H), 4.31 (t, *J* = 4.7 Hz, 2H). ^13^C NMR (100 MHz, CDCl_3_) *δ* ppm = 30.7, 55.8, 60.7, 63.3, 67.4, 171.8. Elemental analysis: calculated (%) = (C_6_H_11_BrO_3_) = C, 34.14; H, 5.25. Experimental (%) = C, 34.09; H, 5.24.

For the synthesis of 3, 2-((ethoxycarbanothioyl)thio) acetic acid (3.85 g, 21.2 mmol, 1.00 equiv.), 2-hydroxyethyl 2-bromoisobutyrate (4.50 g, 21.2 mmol, 1.00 equiv.) and DPTS (6.86 g, 23.3 mmol, 1.10 equiv.) were dissolved in anhydrous dichloromethane (40 mL) under a N_2_ atmosphere. DCC (4.81 g, 23.3 mmol, 1.10 equiv.) was dissolved in anhydrous dichloromethane (10 mL) and transferred, under a N_2_ flow, to the main reaction vessel using a syringe and the reaction was left to stir at ambient temperature for 16 hours. The resulting crude mixture was filtered, diluted in dichloromethane (100 mL) and washed with deionised water (2 × 100 mL) and brine (1 × 100 mL). The organic layer was dried over MgSO_4_. After the removal of DCM *in vacuo*, further purification was performed by automated liquid chromatography (silica gel, eluting a gradient mobile phase increasing in polarity from hexane to hexane : ethyl acetate (70 : 30)). The product, 2-(2-((ethoxycarbonothioyl)thio)acetoxy)ethyl 2-bromoisobutyrate, Xan-Br; 3, was isolated as a yellow oil in 70% yield (5.08 g) and the correct structure was confirmed using ^1^H and ^13^C NMR spectroscopy (ESI Fig. S5†). ^1^H NMR (400 MHz, CDCl_3_) *δ* ppm = 1.43 (t, *J* = 7.1 Hz, 3H), 1.94 (s, 6H), 3.96 (s, 4H), 4.41 (s, 4H), 4.66 (q, *J* = 7.1 Hz, 2H). ^13^C NMR (100 MHz, CDCl_3_) *δ* ppm = 13.70, 30.7, 37.7, 63.0, 63.3, 70.8, 167.8, 171.5, 212.5. Mass spectrometry calculated: [MNa]^+^ (C_11_H_17_BrO_5_S_2_Na) *m*/*z* = 395.28. Experimental: ES MS [MNa]^+^*m*/z = 395. Elemental analysis: calculated (%): (C_11_H_17_BrO_5_S_2_) = C, 35.39; H, 4.59; S, 17.18. Experimental (%) = C, 36.29; H, 4.79; S, 17.06.

### Polymer synthesis – linear polymerisations

For the synthesis of linear *p*(OEGMA) samples, targeting DP_n_ = 50 monomer units, Dod-Br (0.027 g, 0.08 mmol, 1.00 equiv.), PEG_17_-Br (0.25 g, 0.34 mmol, 1.00 equiv.) or Xan-Br, (0.13 g, 0.30 mmol, 1.00 equiv.) along with 2,2′-bipyridyl (0.10 g, 0.64 mmol, 2.00 equiv.) and OEGMA (5.00 g, 16.0 mmol, 50.0 equiv.) were added to a 25 mL round bottom flask, equipped with a magnetic stirrer bar. The mixture was deoxygenated *via* N_2_ bubbling for 30 minutes. A solvent system of IPA/H_2_O (92.5 : 7.5 v/v%, 4.39 : 0.36 mL, 55 wt% w.r.t to monomer, deoxygenated separately *via* N_2_ purge) was added to the reaction vessel and the mixture was deoxygenated by N_2_ bubbling for a further 5 minutes. Anisole (*ca.* 100 μL) was added to the reaction flask for use as an internal standard for determination of monomer conversion by ^1^H NMR. This was calculated from the change in the intensity of OEGMA vinyl peaks during polymerisation, relative to those of the anisole aromatic ^1^H resonances (ESI, Fig. S6†). CuCl (0.032 g, 0.32 mmol, 1.00 equiv.) was added and the reaction vessel was then sealed. The flask was placed into a pre-heated oil bath (40 °C) and left for 24 h after which the polymerisation was stopped by cooling the flask to ambient temperature, exposure to oxygen and dilution with THF. A neutral alumina column was used to remove the copper catalyst, and the solvent was removed *in vacuo*. The crude polymer was purified by precipitation (twice) from acetone into cold petroleum ether (30–40 °C). Residual solvent was removed in a vacuum oven at 40 °C overnight. The purified polymers were characterised by ^1^H NMR in CDCl_3_ and TD-SEC using a DMF/LiBr (0.01 M) eluent.

### Polymer synthesis – branching polymerisations

All branching copolymerisations were conducted using a constant molar ratio of branching co-monomer to initiator of 0.80 to 1.00 ([EGDMA]_0_/[I]_0_ = 0.80) where I = Dod-Br or PEG_17-_Br or Xan-Br. For co-initiated copolymerisations – for example in the preparation of [Dod_*x*_/PEG(_17_)_*y*_]–*p*(OEGMA_50_-*co*-EGDMA_0.80_) or [Dod_*x*_/Xan_*y*_]–*p*(OEGMA_50_-*co*-EGDMA_0.80_), where ∑[I]_0_ = *x* + *y* = 1.00 equivalents – initial molar initiator ratios of [Dod-Br]_0_ : [PEG_17_-Br]_0_ or [Dod-Br]_0_ : [Xan-Br]_0_ were systematically varied to give branched polymers containing controlled chain-end compositions of 1.00 : 0.00, 0.90 : 0.10, 0.75 : 0.25, 0.50 : 0.50, 0.25 : 0.75, 0.10 : 0.90 and 0.00 : 1.00.

In a typical mixed initiated synthesis of [Dod_*x*_/PEG_(17)*y*_]–*p*(OEGMA_50_-*co*-EGDMA_0.80_), targeting DP_n_ = 50 monomer units, where ∑[I]_0_ = *x* + *y* = 0.25 + 0.75 = 1.00 equiv., OEGMA (5.00 g, 16.0 mmol, 50.0 equiv.), EGDMA (0.051 g, 0.32 mmol, 0.80 equiv.), 2,2′-bipyridyl (0.100 g, 0.64 mmol, 2.00 equiv.), Dod-Br (0.027 g, 0.08 mmol, 0.25 equiv.), PEG_17_-Br (0.19 g, 0.25 mmol, 0.75 equiv.) and a solvent system of IPA/H_2_O (92.5 : 7.5 v/v, 4.39 : 0.36 mL, 55 wt% w.r.t to monomer, deoxygenated separately *via* a N_2_ purge) were added to a RBF equipped with a magnetic stirrer bar and all components were deoxygenated *via* N_2_ bubbling for 30 minutes. Anisole (*ca.* 100 μL) was added to the reaction mixture for use as an internal standard for calculation of monomer conversions by ^1^H NMR. CuCl (0.032 g, 0.32 mmol, 1.00 equiv.) was then added, under a positive pressure of N_2_, and the reaction vessel was sealed. Polymerisation and purification procedures were conducted in accordance with the method used for linear polymerisations, described above.

### Polymer synthesis – preparation of thiolated branched copolymers *via* co-initiated ATRP

In a typical synthesis of [Xan_*x*_/Dod_*y*_]–*p*(OEGMA_50_-*co*-EGDMA_0.80_), targeting DP_n_ = 50 monomer units, where ∑[I]_0_ = *x* + *y* = 0.75 + 0.25 = 1.00 equiv., OEGMA (5.00 g, 16.0 mmol, 50.0 equiv.), EGDMA (0.051 g, 0.32 mmol, 0.80 equiv.), 2,2′-bipyridyl (0.100 g, 0.64 mmol, 2.00 equiv.), Dod-Br (0.027 g, 0.08 mmol, 0.25 equiv.), Xan-Br (0.089 g, 0.24 mmol, 0.75 equiv.) were added to a RBF equipped with magnetic stirrer and solvent system IPA/H_2_O (92.5 : 7.5 v/v, 4.39 : 0.36 mL, 55 wt% wrt to monomer, deoxygenated separately *via* N_2_ purge) was added. Anisole (*ca.* 100 μL) was added to the reaction for use as an internal standard for calculation of monomer conversion by ^1^H NMR. CuCl (0.032 g, 0.32 mmol, 1.00 equiv.) was added and the reaction vessel was sealed. Polymerisation and purification processes were conducted in accordance with the method used for linear polymerisations, described above. In a typical deprotection procedure to yield [SH_0.75_/Dod_0.25_]–*p*(OEGMA_50_-*co*-EGDMA_0.80_), the purified copolymer, [Xan_0.75_/Dod_0.25_]–*p*(OEGMA_50_-*co*-EGDMA_0.80_) (0.60 g, 1.66 mmol, 1.00 equiv.) and anhydrous THF (10 mL) were added to a 25 mL round bottom flask equipped with a magnetic stirrer bar. The flask was sealed and deoxygenated *via* a N_2_ purge for 10 minutes. *n*-Butylamine (0.38 mL, 4.15 mmol, 2.50 equiv.) was then added and the reaction was left to stir at ambient temperature. After 1.5 hours, the solvent was removed *in vacuo* and the crude polymer precipitated from acetone into hexane (twice). The pure copolymer was then dried in a vacuum oven at 40 °C for 48 hours.

### Characterisation – lower critical solution temperature measurements

For a typical lower critical solution temperature (LCST) measurement, an aqueous copolymer solution (5 mL, 1 wt%) was prepared in a round bottom flask (10 mL) equipped with a magnetic stirrer bar. The solution was studied through three heating/cooling cycles in an oil bath at a heating/cooling rate of approximately 1 °C min^−1^. The temperatures of copolymer solutions were monitored until the cloud point was observed, *i.e.* when the transparent solution turned opaque, indicating precipitation of the dissolved macromolecules. LCST values were quoted as the mean value (*n* = 3 ± standard deviations).

### Characterisation – surface tensiometry measurements

Aqueous copolymer solutions were prepared at a concentration of 30 wt% in deionised (DI) H_2_O. Serial dilutions of such copolymer solutions in DI H_2_O were used to yield 30 different copolymer concentrations ranging from 6.9 × 10^−9^ to 30 wt%. The use of a 96 well-plate allowed for high throughput analysis of samples; 50 μL of each polymeric surfactant (*n* = 8) was used per well and measured against pure water as the control. Surface tension data was plotted against a log concentration of the surfactant. Critical micelle concentrations (CMC) were calculated by determining the intercept between the lines of best fit obtained from the linear decreases in surface tension (slope) and the lower plateau area.

### Characterisation – contact angle measurements

Aqueous copolymer solutions (5 wt%) were prepared and applied to a glass microscope slide covered with a polytetrafluoroethylene (PTFE) substrate, to create a hydrophobic surface. Aqueous copolymer solutions (5 μL) were applied to the slide and contact angles measured using the static sessile drop method (*n* = 10 ± standard deviations).

### Characterisation – preparation of biosimilar mucus

Carbopol® 940 (0.90 g, 0.90 w/v%) was dissolved in HEPES buffer solution (9.00 mL, 1.30 mM CaCl_2_, 1.00 mM MgSO_4_ and 137 mM NaCl, pH 7.4) using magnetic stirring. A lipid mixture of phosphatidylcholine (0.018 g, 0.18% weight/volume (w/v)%), cholesterol (0.0036 g, 0.36 w/v%), polysorbate 80 (0.033 g, 4 : 1 ratio) and HEPES buffer solution (1 mL) was prepared and added to the Carbopol solution when approximately 90% of the Carbopol had dissolved. Mucin from porcine stomach (0.50 g, 5.00 w/v%) was added to the solution and the pH altered to 7.4 with the addition of NaOH (1 M), which was monitored using a pH probe. BSA (0.31 g, 3.10 w/v%) was added and the pH was once again adjusted to 7.4. Biosimilar mucus was stored overnight at 2–4 °C before use and discarded if not used within a maximum of four days from the day of preparation.

### Preparation and characterisation of O/W emulsions

O/W emulsions were prepared using a 1 : 1 v/v ratio of oil : water, using either *n*-dodecane or squalene as the oil phase. Aqueous copolymer solutions (3 mL, 5 mg mL^−1^) and *n*-dodecane or squalene (3 mL) were added to a glass vial (14 mL); the mixture was then homogenised using an IKA T 25 ULTRA-TURRAX over-head high-shear homogeniser at 24 000 rpm for 2 minutes. Emulsions were stored overnight in a sealed glass vial at ambient temperature before characterisation *via* laser diffraction. O/W emulsions were added dropwise to the dispersion unit containing approximately 100 mL deionised water (DI H_2_O) at a stirring rate of 1000 rpm at ambient temperature. The volume-average droplet diameters (*D*_[4,3]_) are quoted as an average of ≥20 measurements, where *D*_[4,3]_ = ∑*D*_*i*_^4^*N*_*i*_/∑*D*_*i*_^3^N_*i*_. For further stability studies, emulsions were stored in sealed glass vials at ambient temperature and were re-assessed by laser diffraction and optical microscopy at the time intervals stated.

### Mucosal trigged emulsion release studies

For mucosal triggered release studies emulsions were prepared as above with the inclusion of either Oil Red O or Oil Blue A (0.1 wt% with respect to the oil phase) as a hydrophobic drug mimic. In a typical mucosal triggered release study, biosimilar mucus was spread onto a glass microscope slide. Polymeric emulsifier-stabilised O/W emulsions (100 μL) were applied to the centre of the glass slide. The slides were then imaged using optical microscopy both immediately after application of the O/W emulsion and again 10 minutes after the addition.

## Conflicts of interest

SEE and SPR are co-inventors of a recently filed patent application that describes the work reported here.

## Supplementary Material

RA-010-D0RA05820C-s001
