# Supplementary material for: Mucus-responsive functionalized emulsions: design, synthesis and study of novel branched polymers as functional emulsifiers
Source: RSC Adv. 2020 Aug 18;10(51):30463–75. doi: 10.1039/d0ra05820c (PMC9056332; doi:10.1039/d0ra05820c)
Supplement: RA-010-D0RA05820C-s001 [file RA-010-D0RA05820C-s001.pdf]

Electronic Supplementary Material (ESI) for RSC Advances.
This journal is © The Royal Society of Chemistry 2020

Electronic Supplementary Information

Mucus-responsive functionalized emulsions: Design, synthesis and study of novel branched polymers as functional emulsifiers.

Stephanie E. Edwards[1], Sean Flynn[2], James J. Hobson[1], Pierre Chambon[2], Helen Cauldbeck[1] and Steve P. Rannard[2]*

[1]Department of Chemistry, University of Liverpool, Crown Street, L69 7ZD, UK

[2]Materials Innovation Factory, University of Liverpool, Oxford Street, Liverpool L7 3NY, UK

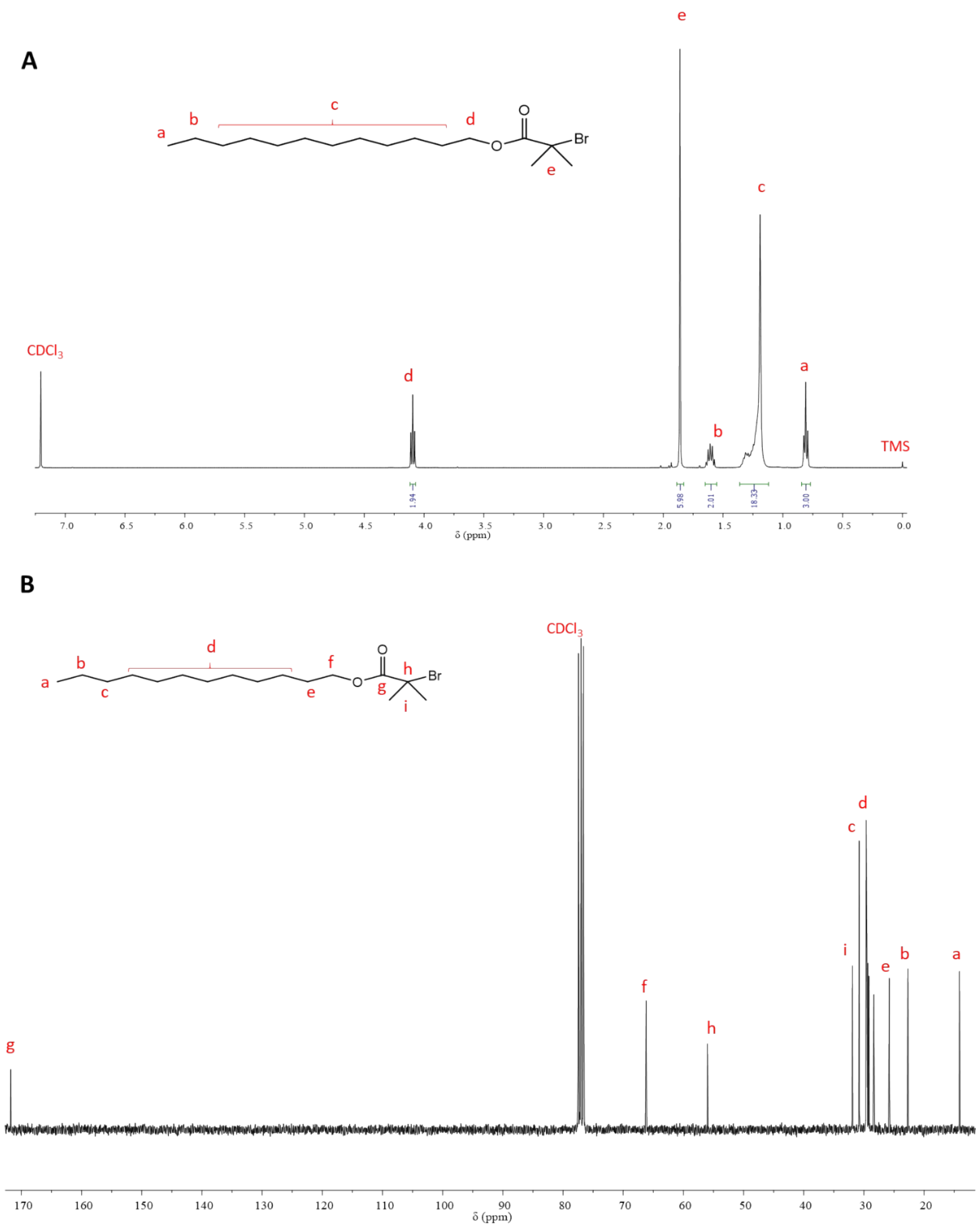

Figure S1 NMR spectra of Dod-Br A) ^{1}H NMR ($CDCl_3$, 400 MHz) and B) ^{13}C NMR ($CDCl_3$, 100 MHz).

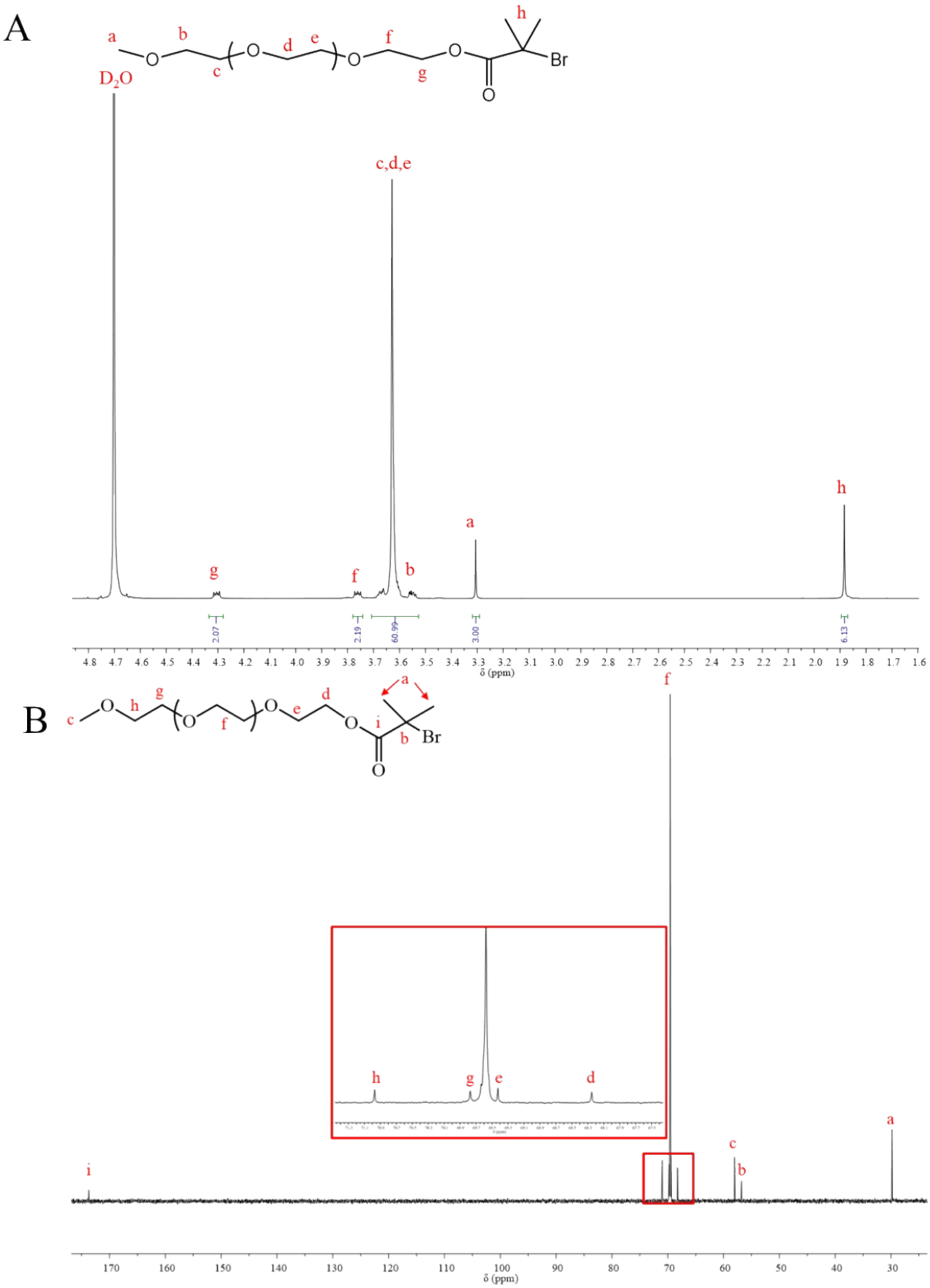

Figure S2 NMR spectra of purified PEG_{17}-Br; A) ^{1}H NMR (D_2O, 400 MHz) and B) ^{13}C NMR (D_2O, 100 MHz).

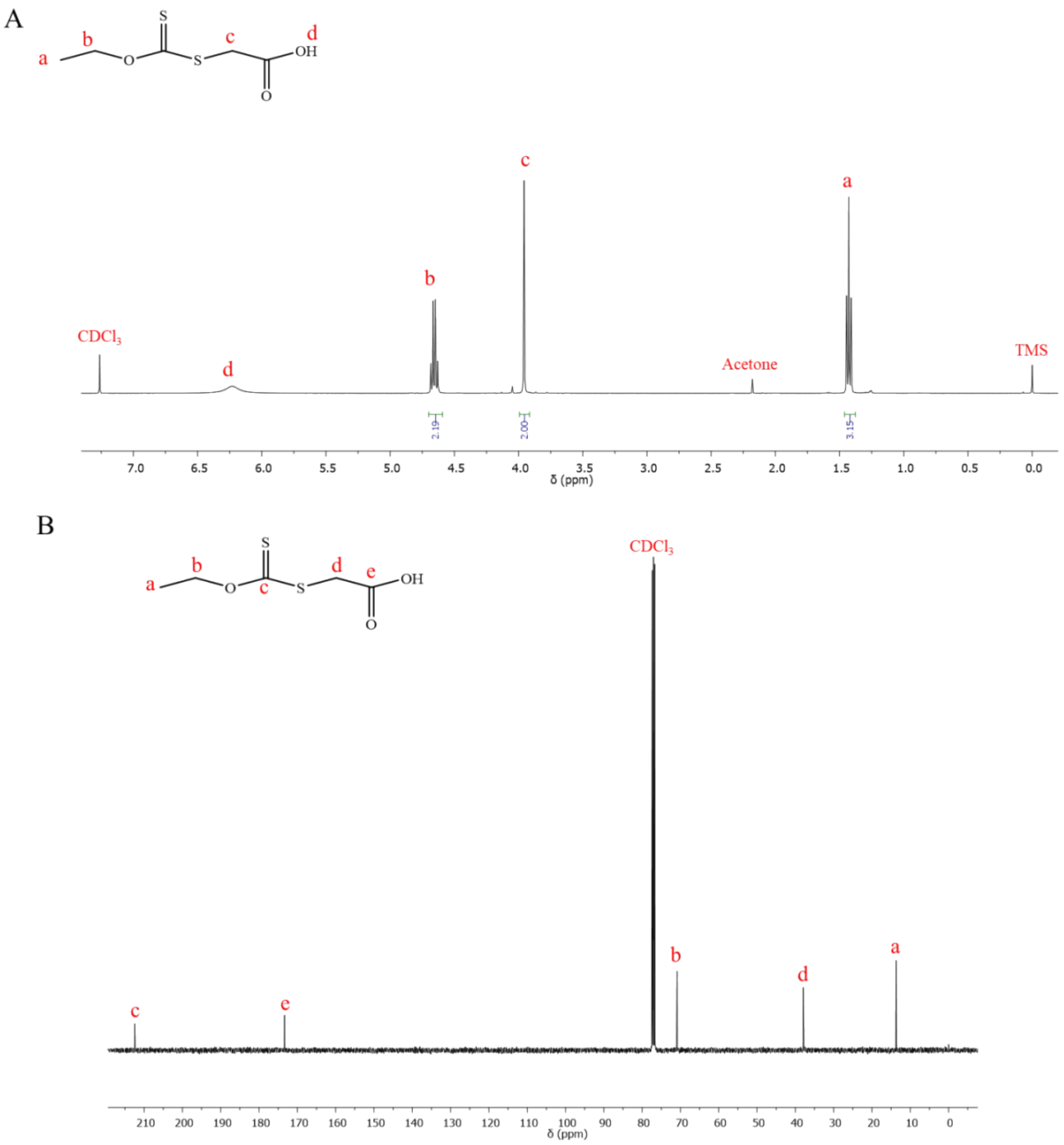

Figure S3 NMR spectra of 2-((Ethoxycarbanothioyl)thio) acetic acid; A) ^{1}H NMR ($CDCl_3$, 400 MHz) and B) ^{13}C NMR ($CDCl_3$, 100 MHz).

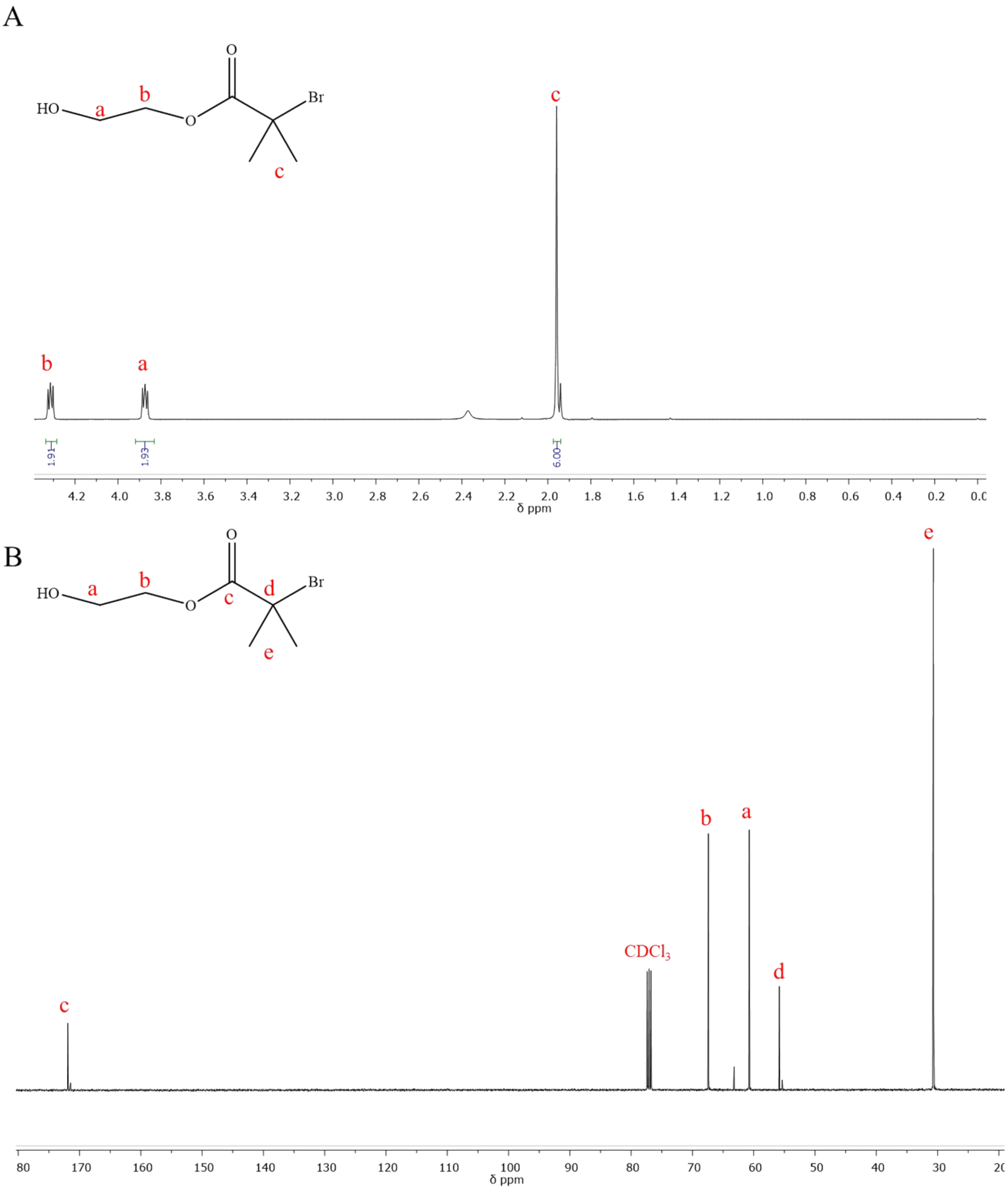

Figure S4 NMR spectra of 2-Hydroxyethyl 2-Bromoisobutyrate; A) ^{1}H NMR ($CDCl_3$, 400 MHz) and B) ^{13}C NMR ($CDCl_3$, 100 MHz).

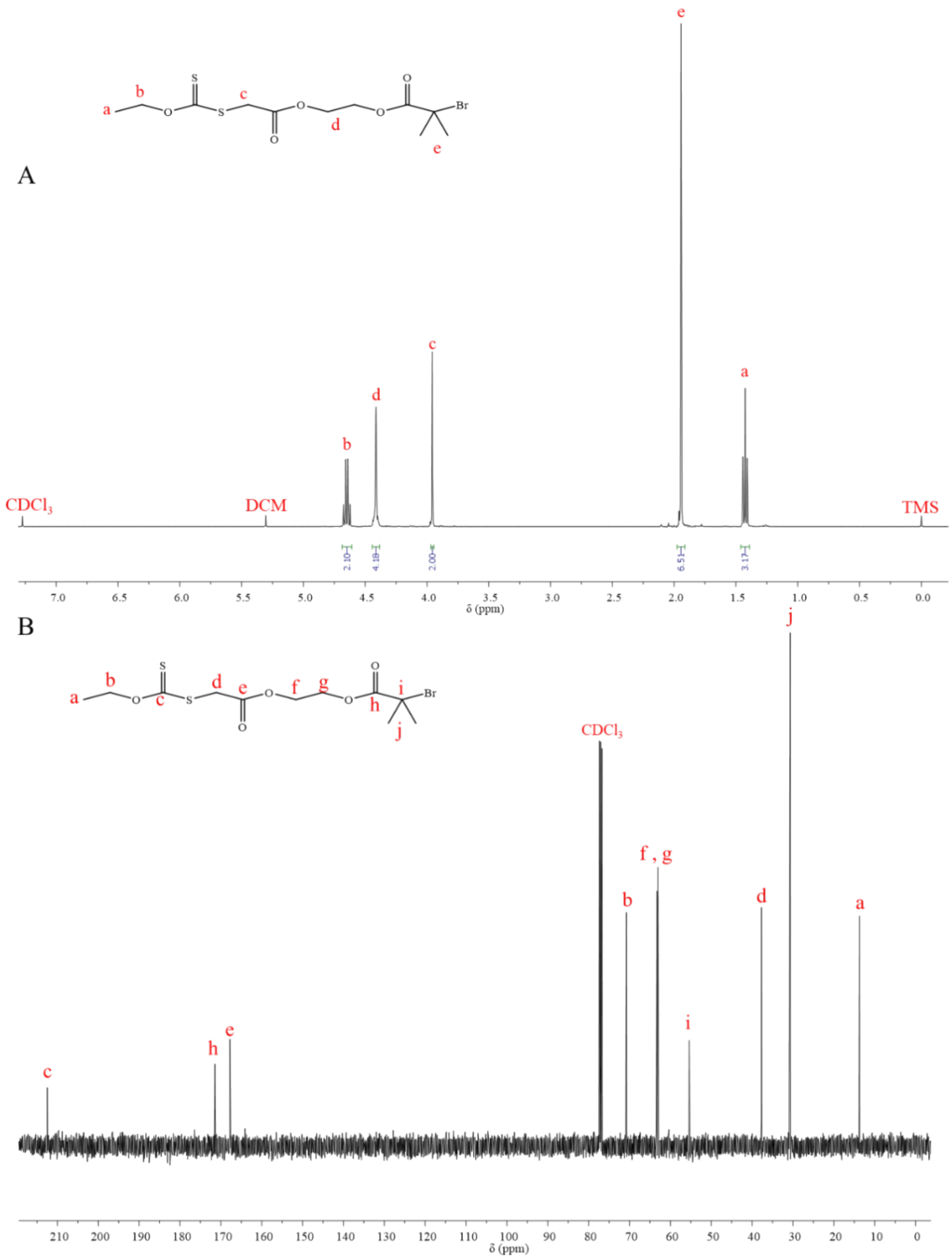

Figure S5 NMR spectra of Xan-Br. A) ^1H NMR ($CDCl_3$, 400 MHz) and B) ^{13}C NMR ($CDCl_3$, 100 MHz.

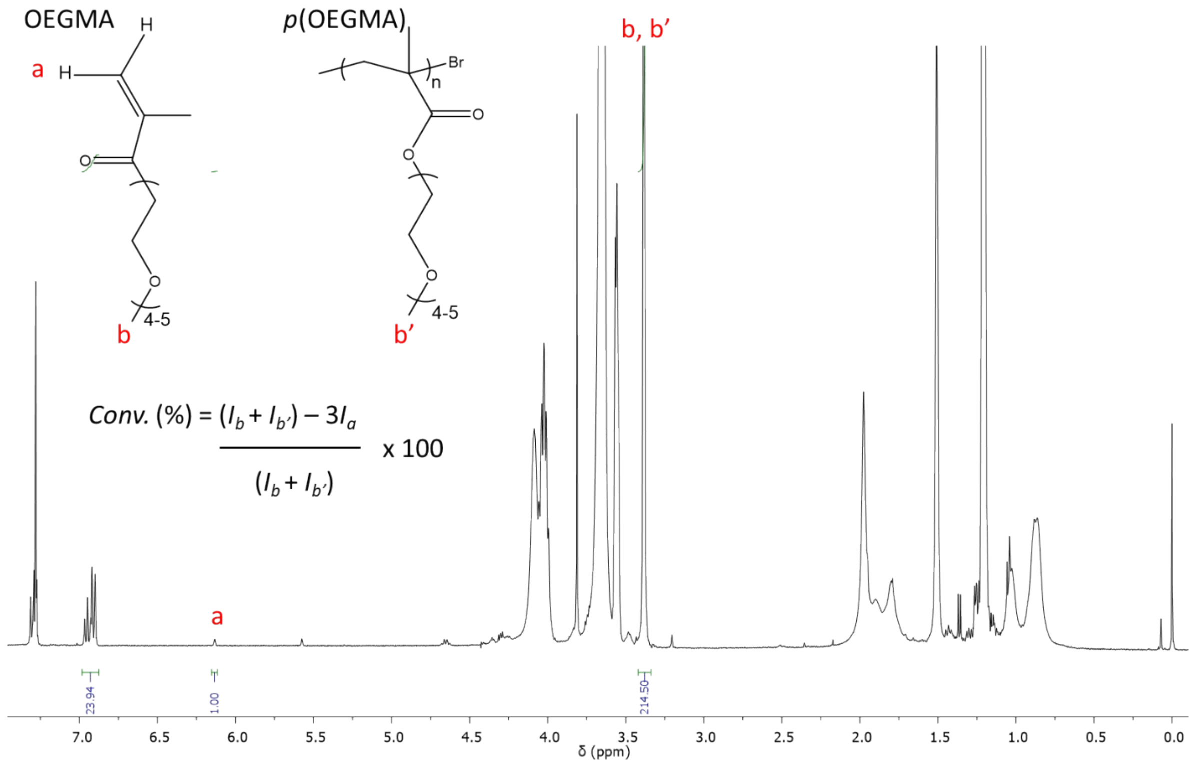

Figure S6 Quantification of the monomer conversion achieved in the polymerisation of OEGMA by ^{1}H NMR analysis of the reaction mixture after 19 hours ($CDCl_3$, 400 MHz).

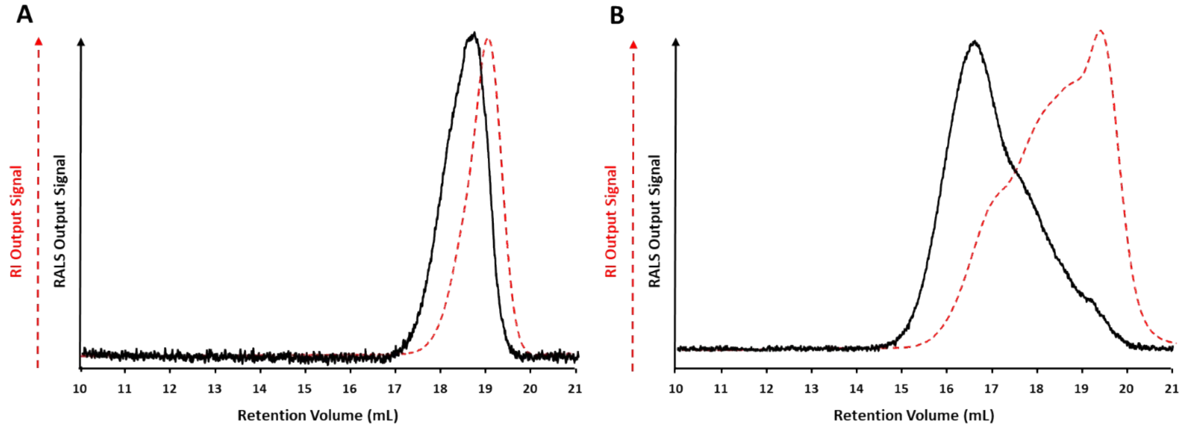

Figure S7 Comparisons of linear homopolymers and branched copolymers by TD-SEC at 60 °C in DMF/0.01 M LiBr. Overlays of refractive index (RI, red dashed lines) and right-angle light scattering (RALS, solid black lines) chromatograms for A) linear [$Dod_{1.00}$]-*p*($OEGMA_{50}$) and B) branched equivalent [$Dod_{1.00}$]-*p*($OEGMA_{50}$-*co*-$EGDMA_{0.80}$).

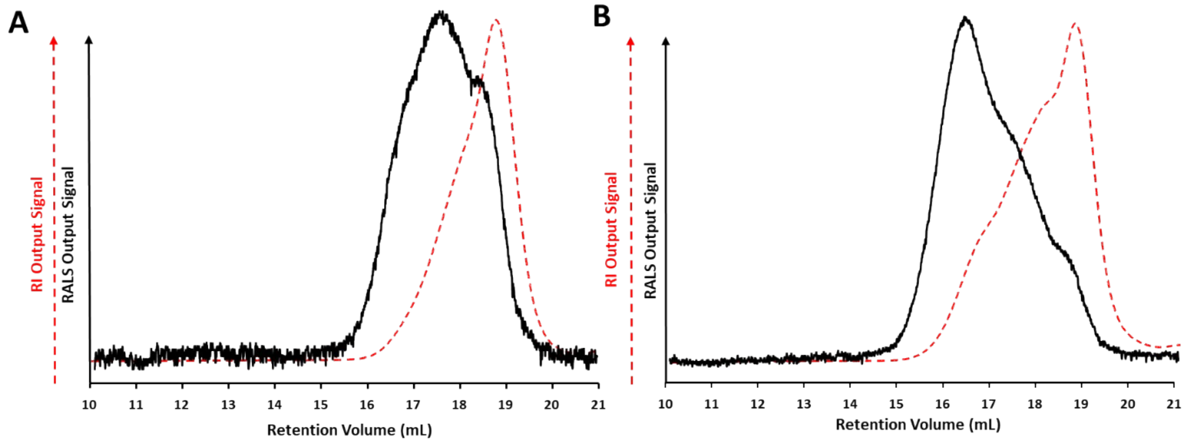

Figure S8 Comparisons of linear homopolymers and branched copolymers by TD-SEC at 60 °C in DMF/0.01 M LiBr. Overlays of refractive index (RI, red dashed lines) and right-angle light scattering (RALS, solid black lines) chromatograms for A) linear [$PEG_{(17)1.00}$]-*p*($OEGMA_{50}$) and B) branched equivalent $PEG_{(17)1.00}$-*p*($OEGMA_{50}$-*co*-$EGDMA_{0.80}$).

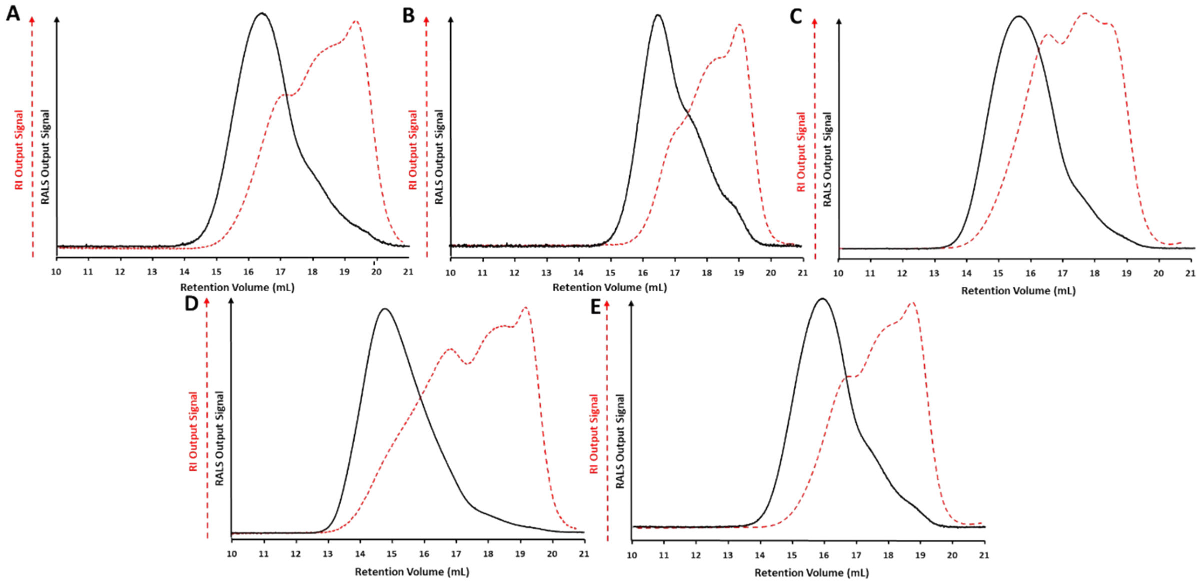

Figure S9 TD-SEC analysis of branched copolymers containing mixed chain-end functionality. Overlaid RI (dashed red lines) and RALS (solid black lines) chromatograms obtained from TD-SEC of branched copolymers with general composition of [Dod_x/$PEG_{(17)y}$]-*p*($OEGMA_{50}$-*co*-$EGDMA_{0.80}$), where x + y = 1.00. A) [$Dod_{0.90}$/$PEG_{(17)0.10}$]-*p*($OEGMA_{50}$-*co*-$EGDMA_{0.80}$), B) [$Dod_{0.75}$/$PEG_{(17)0.25}$]-*p*($OEGMA_{50}$-*co*-$EGDMA_{0.80}$), C) [$Dod_{0.50}$/$PEG_{(17)0.50}$]-*p*($OEGMA_{50}$-*co*-$EGDMA_{0.80}$), D) [$Dod_{0.25}$/$PEG_{(17)0.75}$]-*p*($OEGMA_{50}$-*co*-$EGDMA_{0.80}$) and E) [$Dod_{0.10}$/$PEG_{(17)0.90}$]-*p*($OEGMA_{50}$-*co*-$EGDMA_{0.80}$)

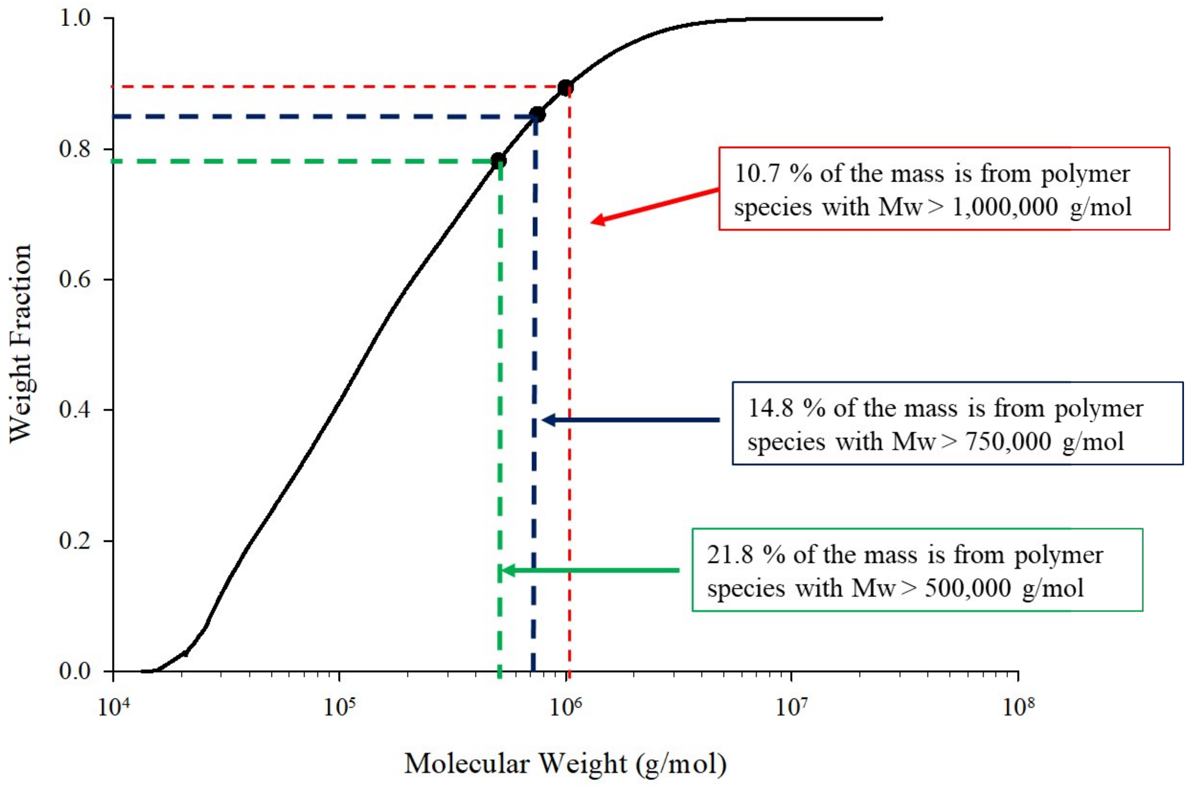

Figure S10 Exemplary cumulative weight fraction analysis of branched copolymer [$Dod_{0.25}$/$PEG_{(17)0.75}$]-*p(*$OEGMA_{50}$-*co*-$EGDMA_{0.80}$). This specifically highlights the weight fraction of branched copolymer species with absolute molecular weights (M) >500,000 g mol^{-1} (green dashed lines), >750,000 g mol^{-1} (blue dashed lines) and >1,000,000 g mol^{-1} (red dashed lines). TD-SEC analysis conducted at 60 °C using a DMF/0.01 M LiBr eluent.

Table S1 Quantification of the contributions of high molecular weight species present within the molecular weight distributions to the overall mass of branched copolymers with the general formula [Dod_x/$PEG_{(17)y}$]-*p(*$OEGMA_{50}$-*co*-$EGDMA_{0.80}$). TD-SEC analysis conducted at 60 °C using a DMF/0.01 M LiBr eluent.

**Branched copolymer**	**Weight %** > **500 kg mol^{-1}**	**Weight %** > **750 kg mol^{-1}**	**Weight %** > **1, 000 kg mol^{-1}**
[$Dod_{1.00}$]-*p*($OEGMA_{50}$-*co*-$EGDMA_{0.80}$)	11.2	2.8	2.5
[$Dod_{0.90}$/$PEG_{(17)0.10}$]-*p*($OEGMA_{50}$-*co*-$EGDMA_{0.80}$)	14.2	8.5	5.4
[$Dod_{0.75}$/$PEG_{(17)0.25}$]-*p*($OEGMA_{50}$-*co*-$EGDMA_{0.80}$)	15.6	9.1	5.9
[$Dod_{0.50}$/$PEG_{(17)0.50}$]-*p*($OEGMA_{50}$-*co*-$EGDMA_{0.80}$)	5.9	2.19	0.9
[$Dod_{0.25}$/$PEG_{(17)0.75}$]-*p*($OEGMA_{50}$-*co*-$EGDMA_{0.80}$)	21.8	14.8	10.7
[$Dod_{0.10}$/$PEG_{(17)0.90}$]-*p*($OEGMA_{50}$-*co*-$EGDMA_{0.80}$)	33.7	27.2	2.3
[$PEG_{(17)1.00}$]-*p*($OEGMA_{50}$-*co*-$EGDMA_{0.80}$)	6.4	2.9	1.4

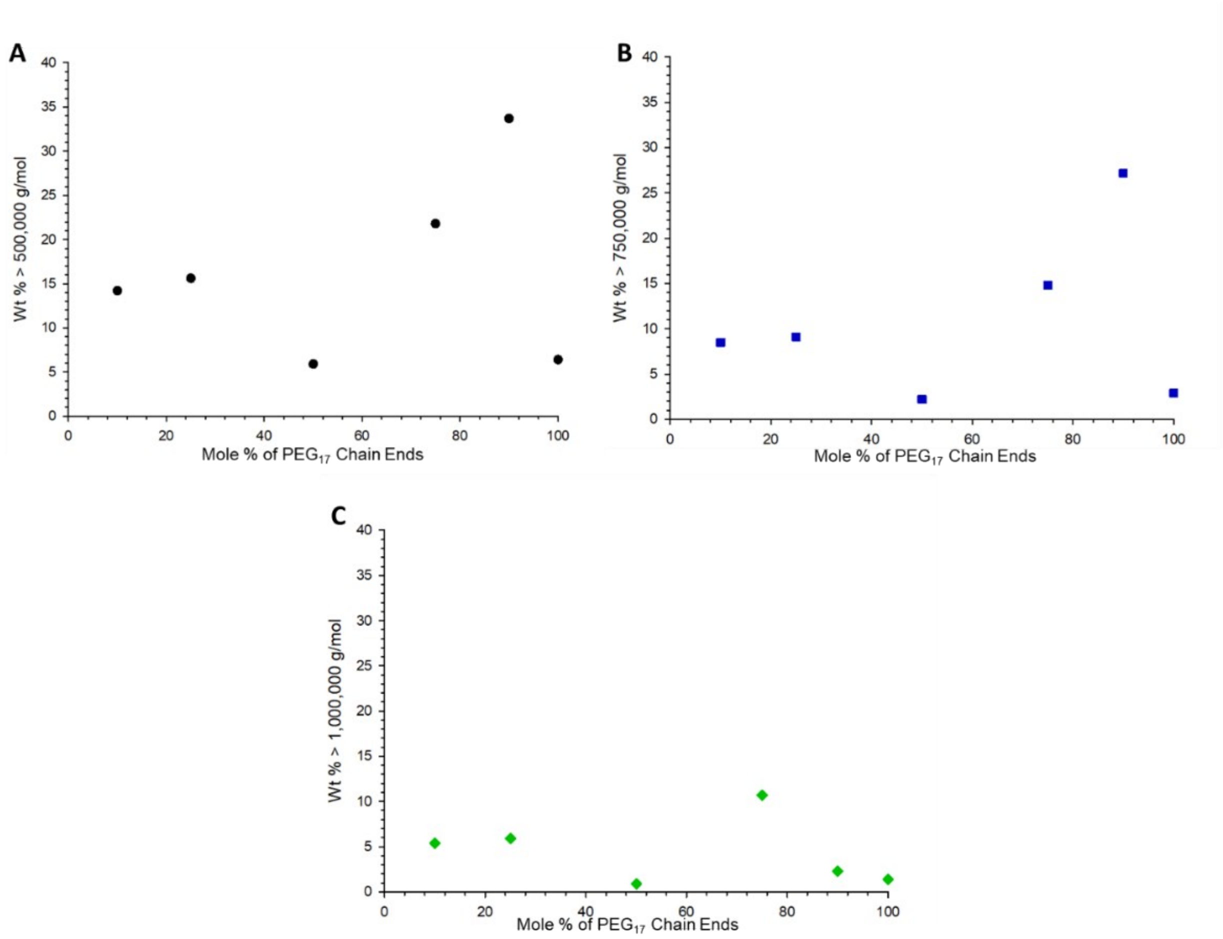

Figure S11 Variation in the weight percentage of high molecular weight species within branched copolymer molecular weight distributions across the [Dod_x/$PEG_{(17)y}$]-*p(*$OEGMA_{50}$-*co*-$EGDMA_{0.80}$) series. Plots of the weight percent of branched copolymer species with molecular weights greater than A) 500,000 g mol^{-1} B) 750,000 g mol^{-1} and C) 1,000,000 g mol^{-1} against the molar percentage of PEG_{17}-Br initiator used in the copolymerisation.

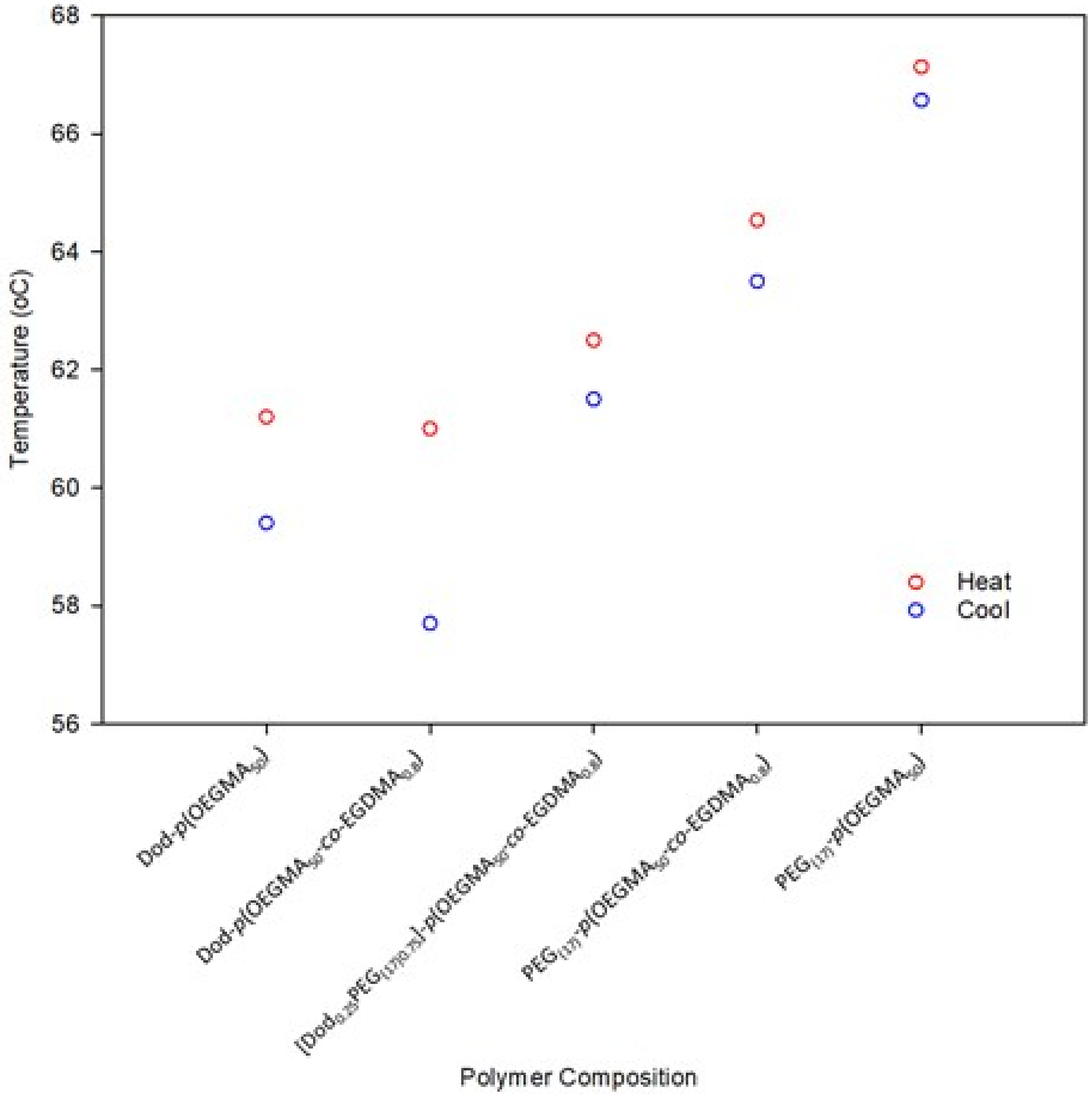

Figure S12 Aqueous lower critical solution temperature behaviour of linear homopolymers and branched copolymers containing varied chain-end compositions.

Table S2 Contact angles measured for aqueous solutions of linear homopolymers and branched copolymers containing varying ratios of Dod and PEG_{17} chain end compositions (5 wt %) on a PTFE surface.

Polymer	Contact Angle (°)		
	**Mean**	**Median**	**Std. Dev. (±)**
[$Dod_{1.00}$]-*p*($OEGMA_{50}$)	83.94	84.12	2.30
[$Dod_{1.00}$]-*p*($OEGMA_{50}$-*co*-$EGDMA_{0.80}$)	79.59	79.38	2.16
[$Dod_{0.90}$/$PEG_{(17)0.10}$]-*p*($OEGMA_{50}$-*co*-$EGDMA_{0.80}$)	102.65	103.53	2.79
[$Dod_{0.75}$/$PEG_{(17)0.25}$]-*p*($OEGMA_{50}$-*co*-$EGDMA_{0.80}$)	102.93	101.89	5.06
[$Dod_{0.50}$/$PEG_{(17)0.50}$]-*p*($OEGMA_{50}$-*co*-$EGDMA_{0.80}$)	102.92	101.89	3.77
[$Dod_{0.10}$/$PEG_{(17)0.90}$]-*p*($OEGMA_{50}$-*co*-$EGDMA_{0.80}$)	87.26	89.76	11.4
[$PEG_{(17)1.00}$]-*p*($OEGMA_{50}$)	106.44	106.97	4.05
[$PEG_{(17)1.00}$]-*p*($OEGMA_{50}$-*co*-$EGDMA_{0.80}$)	91.26	91.15	4.94
DI H_2O	124.65	125.99	3.26

Table S3 Critical micelle concentrations (CMC) and minimum surface tension (ST_{min}) values determined for polymers and branched copolymers at 20 °C. Values are expressed as mg/L and errors are expressed as a graphical percentage error.

**Polymer**	**Initial CMC (mg L^{-1})**	**ST_{min} (mg L^{-1})**
[$Dod_{1.00}$]-*p*($OEGMA_{50}$)	4.72×10^{-8} (± 8%)	9.30×10^{-3}
[$PEG_{(17)1.00}$]-*p*($OEGMA_{50}$)	7.40×10^{-8} (± 2 %)	9.30×10^{-3}
[$Dod_{1.00}$]-*p*($OEGMA_{50}$-*co*-$EGDMA_{0.80}$)	2.36×10^{-6} (± 10 %)	7.50×10^{-2}
[$Dod_{0.90}$/$PEG_{(17)0.10}$]-*p*($OEGMA_{50}$-*co*-$EGDMA_{0.80}$)	2.79×10^{-8} (± 7 %)	7.30×10^{-5}
[$Dod_{0.75}$/$PEG_{(17)0.25}$]-*p*($OEGMA_{50}$-*co*-$EGDMA_{0.80}$)	4.74×10^{-8} (± 2 %)	2.92×10^{-4}
[$Dod_{0.50}$/$PEG_{(17)0.50}$]-*p*($OEGMA_{50}$-*co*-$EGDMA_{0.80}$)	1.08×10^{-8} (± 16 %)	9.30×10^{-3}
[$Dod_{0.10}$/$PEG_{(17)0.90}$]-*p*($OEGMA_{50}$-*co*-$EGDMA_{0.80}$)	2.35×10^{-8} (± 17 %)	1.46×10^{-4}
[$PEG_{(17)1.00}$]-*p*($OEGMA_{50}$-*co*-$EGDMA_{0.80}$)	7.25×10^{-8} (± 0.7 %)	7.50×10^{-2}

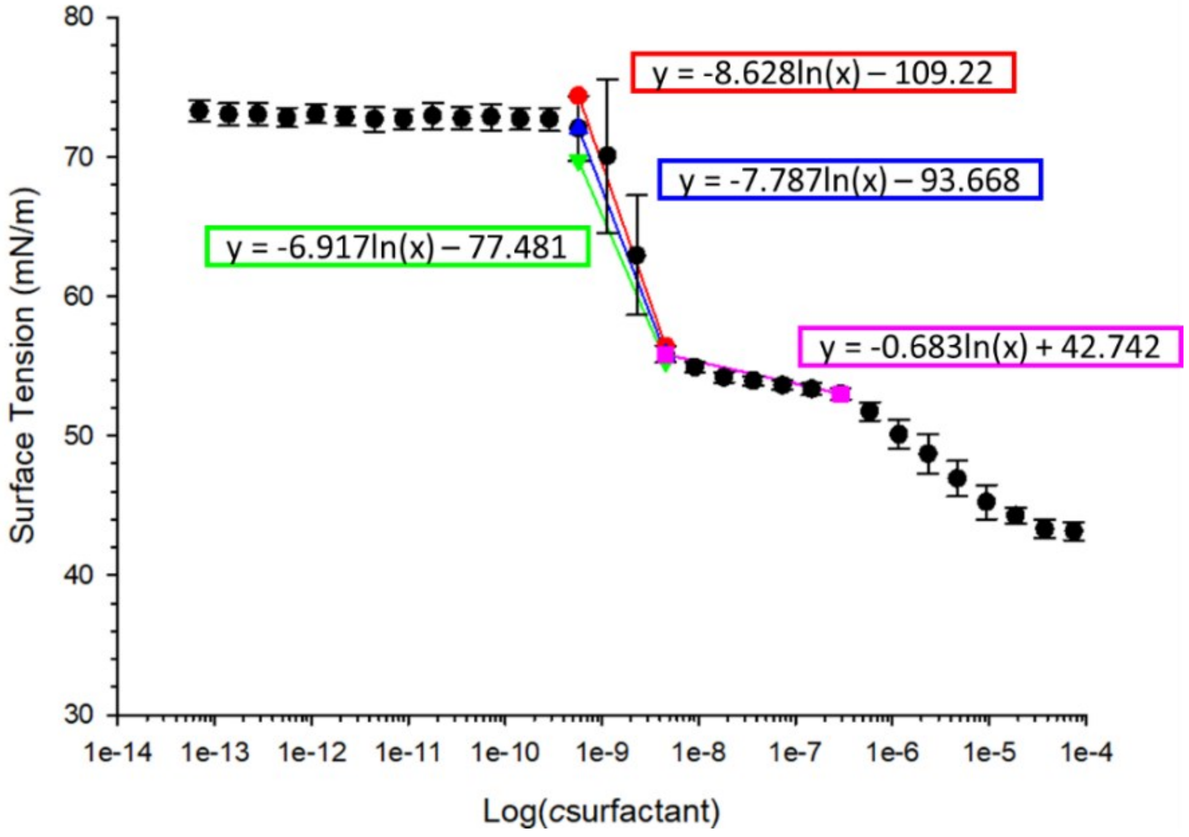

Figure S13 An example of the determination of the CMC of an aqueous (co)polymer solution from a plot of Surface Tension (ST) against (co)polymer concentration. The CMC value was obtained by calculating lines of best fit for the linear decrease in surface tension (slope, blue) and the lower plateau area (purple), and rearrangement of the resulting equation to determine the intercept (x). To calculate graphical percentage error for CMC measurements, the minimum and maximum gradient was determined by plotting the minimum (green) and maximum (red) lines of best fit.

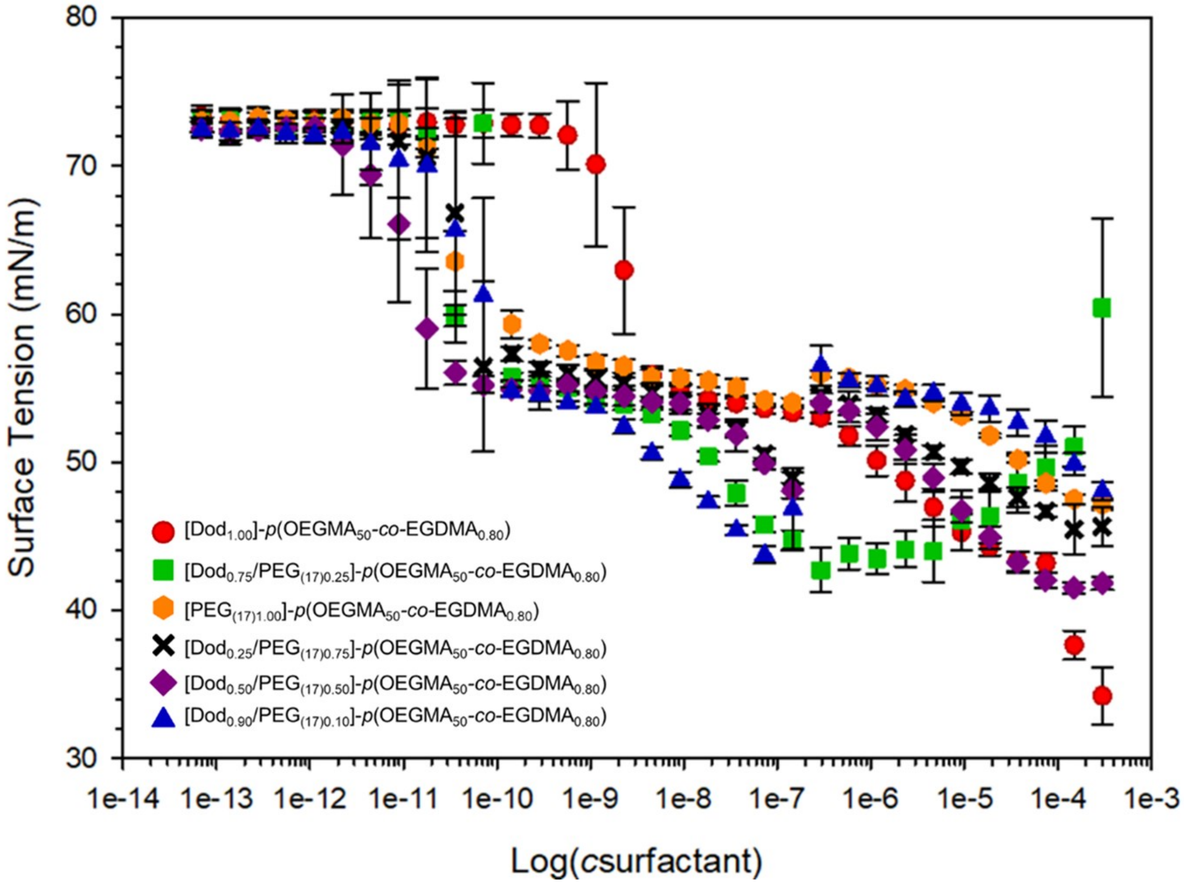

Figure S14 CMC behaviour of branched copolymers containing varied chain-end compositions. Overlaid plots of ST against branched copolymer concentration for: [$Dod_{1.00}$]-*p*($OEGMA_{50}$-*co*-$EGDMA_{0.80}$) (red circles), [$Dod_{0.90}$/$PEG_{(17)0.10}$]-*p*($OEGMA_{50}$-*co*-$EGDMA_{0.80}$) (Blue triangles), [$Dod_{0.75}$/$PEG_{(17)0.25}$]-*p*($OEGMA_{50}$-*co*-$EGDMA_{0.80}$) (green squares), [$Dod_{0.50}$/$PEG_{(17)0.50}$]-*p*($OEGMA_{50}$-*co*-$EGDMA_{0.80}$) (purple diamonds), [$Dod_{0.25}$/$PEG_{(17)0.75}$]-*p*($OEGMA_{50}$-*co*-$EGDMA_{0.80}$) (black crosses) and [$PEG_{(17)1.00}$]-*p*($OEGMA_{50}$-*co*-$EGDMA_{0.80}$) (orange hexagons).

Table S4 Analysis of the size and stability of dodecane-in-water emulsions stabilised by branched copolymer emulsifiers containing varied chain-end functionality. Volume average mean diameters ($D_{[4,3]}$) of dodecane-in-water emulsions over a 40-day time period.

Polymer	$D_{[4,3]}$ (µm)				
	**Day 1**	**Day 7**	**Day 10**	**Day 20**	**Day 40**
[$Dod_{1.00}$]-*p*($OEGMA_{50}$-*co*-$EGDMA_{0.80}$)	11.9	12.8	13.5	13.1	13.5
[$Dod_{0.90}$/$PEG_{(17)0.10}$]-*p*($OEGMA_{50}$-*co*-$EGDMA_{0.80}$)	12.8	13.1	13.9	14.3	13.8
[$Dod_{0.75}$/$PEG_{(17)0.25}$]-*p*($OEGMA_{50}$-*co*-$EGDMA_{0.80}$)	14.5	14.1	15.5	16.2	15.6
[$Dod_{0.50}$/$PEG_{(17)0.50}$]-*p*($OEGMA_{50}$-*co*-$EGDMA_{0.80}$)	15.3	15.5	15.5	14.8	15.2
[$PEG_{(17)1.00}$]-*p*($OEGMA_{50}$-*co*-$EGDMA_{0.80}$)	16.9	-*	-*	-*	-*

* $D_{[4,3]}$ could not be obtained due to complete demulsification of the [$PEG_{(17)1.00}$]-*p*($OEGMA_{50}$-*co*-$EGDMA_{0.80}$)-stabilised dodecane-in-water emulsion before the stated time point.

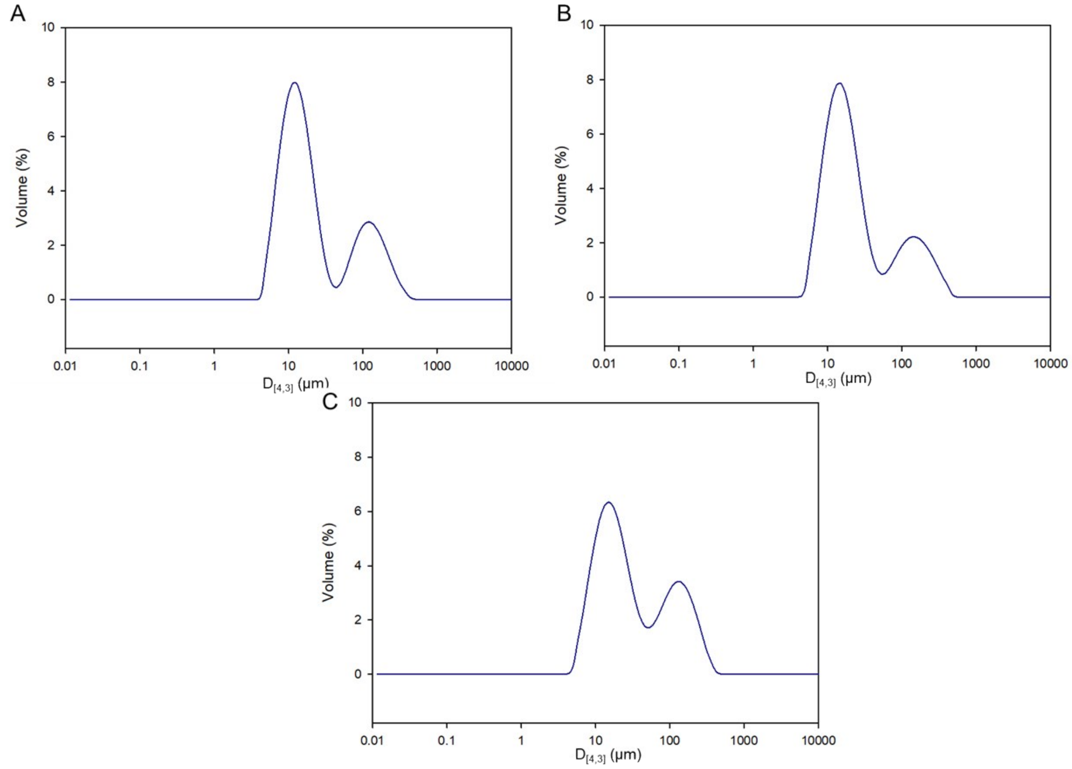

Figure S15 Volume-average size distributions obtained by laser-diffraction of dodecane-in-water emulsions, stabilised using a mixed emulsifier system of [$Dod_{1.00}$]-*p*($OEGMA_{50}$) and [$PEG_{(17)1.00}$]-*p*($OEGMA_{50}$) linear homopolymers with mass fractions of: A) 0.75: 0.25, B) 0.50: 0.50 and C) 0.25: 0.75 respectively.

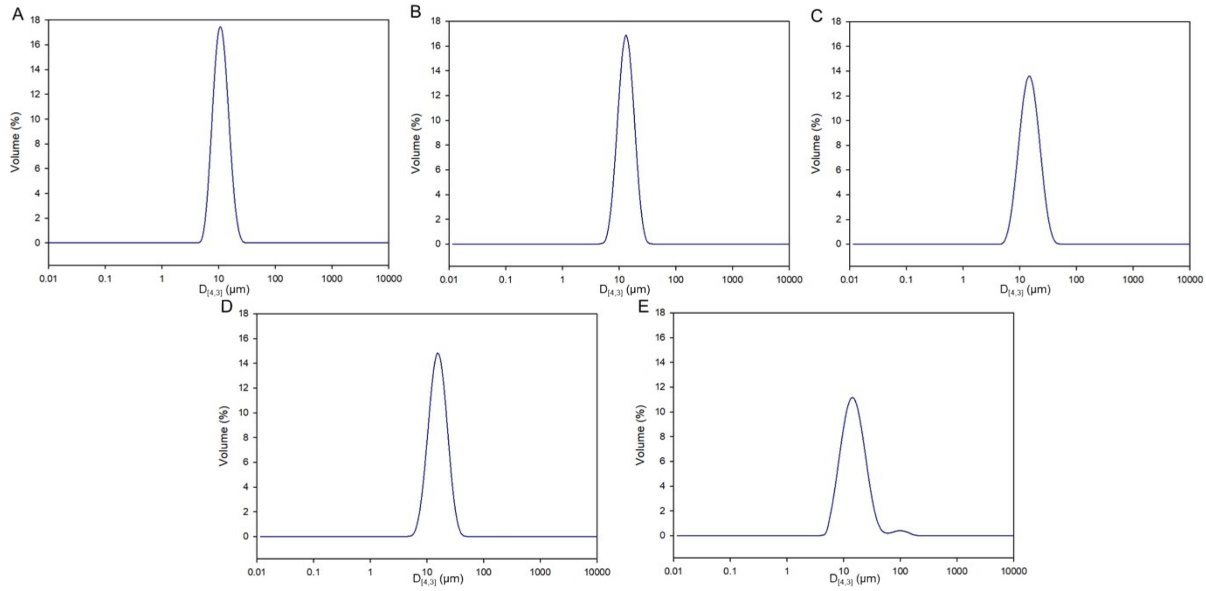

Figure S16 Volume-average size distributions of dodecane-in-water emulsion droplets stabilised by branched copolymer emulsifiers obtained by laser-diffraction. A) [Dod$_{1.00}$]-*p*(OEGMA$_{50}$-*co*-EGDMA$_{0.80}$), B) [Dod$_{0.75}$/PEG$_{(17)0.25}$]-*p*(OEGMA$_{50}$-*co*-EGDMA$_{0.80}$), C) [Dod$_{0.50}$/PEG$_{(17)0.50}$]-*p*(OEGMA$_{50}$-*co*-EGDMA$_{0.80}$), D) [Dod$_{0.25}$/PEG$_{(17)0.75}$]-*p*(OEGMA$_{50}$-*co*-EGDMA$_{0.80}$) and E) [PEG$_{(17)1.00}$]-*p*(OEGMA$_{50}$-*co*-EGDMA$_{0.80}$).

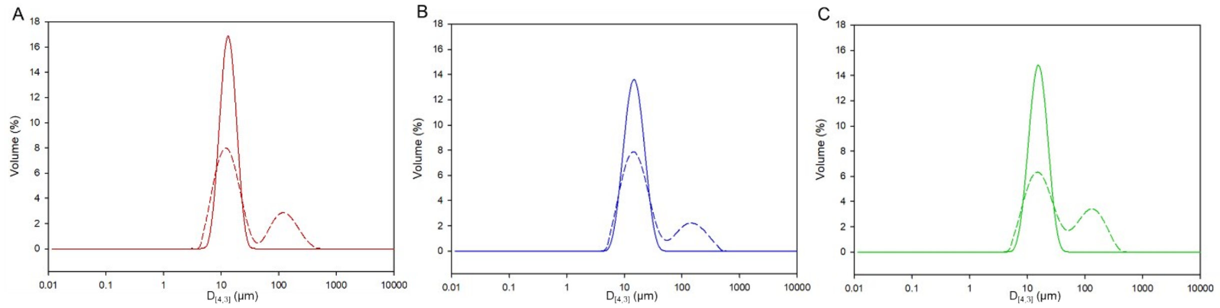

Figure S17 The impact of emulsifier architecture on emulsion droplet size. Overlaid size distributions obtained by laser diffraction of emulsion droplets stabilised by A) [Dod$_{0.75}$/PEG$_{(17)0.25}$]-*p*(OEGMA$_{50}$-*co*-EGDMA$_{0.80}$) (solid red lines) and [Dod$_{1.00}$]-*p*(OEGMA$_{50}$)/[PEG$_{(17)1.00}$]-*p*(OEGMA$_{50}$) (75/25 wt %, dashed red lines), B) [Dod$_{0.50}$/PEG$_{(17)0.50}$]-*p*(OEGMA$_{50}$-*co*-EGDMA$_{0.80}$) (solid blue lines) and [Dod$_{1.00}$]-*p*(OEGMA$_{50}$)/[PEG$_{(17)1.00}$]-*p*(OEGMA$_{50}$) (50/50 wt %, dashed blue lines) and C) [Dod$_{0.25}$/PEG$_{(17)0.75}$]-*p*(OEGMA$_{50}$-*co*-EGDMA$_{0.80}$) (solid green lines) and [Dod$_{1.00}$]-*p*(OEGMA$_{50}$)/[PEG$_{(17)1.00}$]-*p*(OEGMA$_{50}$) (25/75 wt %, dashed green lines).

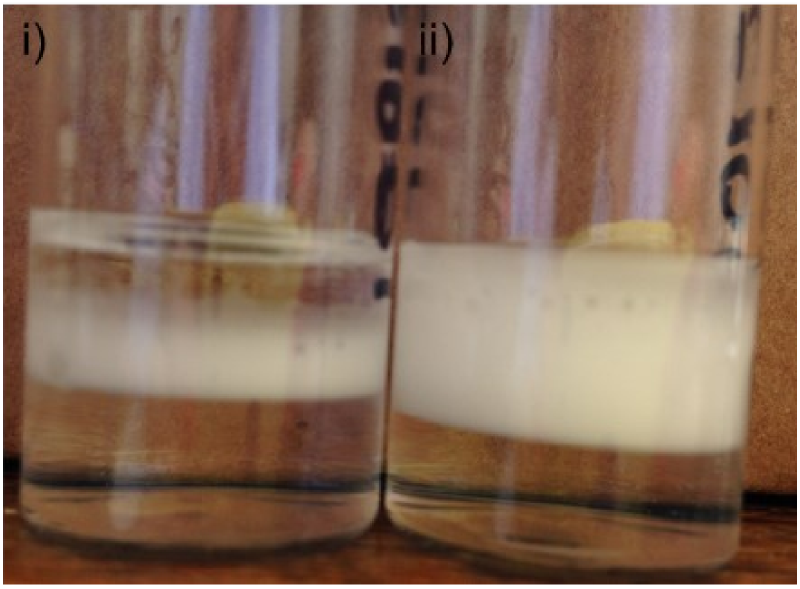

Figure S18 Photographic evidence of the demulsification of a dodecane-in-water emulsion after 72 hours stabilised with i) [PEG$_{(17)1.00}$]-*p*(OEGMA$_{50}$) (left) and ii) [PEG$_{(17)1.00}$]-*p*(OEGMA$_{50}$-*co*-EGDMA$_{0.80}$) (right).

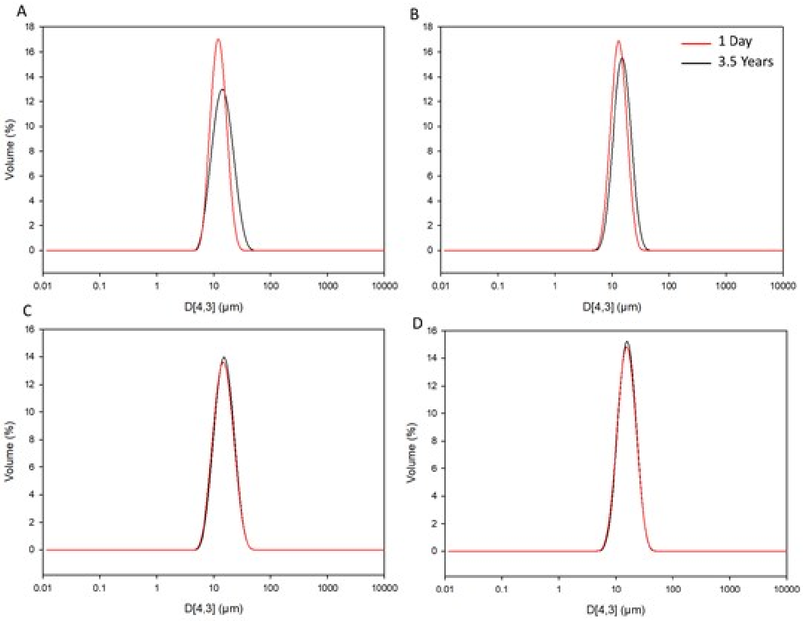

Figure S19 Laser diffraction analysis of dodecane-in-water emulsions stored for 1 day and 3 years at ambient temperature, stabilised with polymeric emulsifiers: A) Dod$_{1.00}$-*p*(OEGMA$_{50}$-*co*-EGDMA$_{0.80}$, B) [Dod$_{0.75}$/PEG$_{(17)0.25}$]-*p*(OEGMA$_{50}$-*co*-EGDMA$_{0.80}$), C) [Dod$_{0.50}$/PEG$_{(17)0.50}$]-*p*(OEGMA$_{50}$-*co*-EGDMA$_{0.80}$), D) [Dod$_{0.25}$/PEG$_{(17)0.75}$]-*p*(OEGMA$_{50}$-*co*-EGDMA$_{0.80}$).

Table S5 Analysis of the size and stability of dodecane-in-water emulsions stabilised by branched copolymer emulsifiers containing varied chain-end functionality. Volume average mean diameters ($D_{[4,3]}$) of dodecane-in-water emulsions over a 3-year time period.

**Polymer**	**$D_{[4,3]}$ (µm)**	
	**+24 hours**	**+ 3 years**
[$Dod_{1.00}$]-*p*($OEGMA_{50}$-*co*-$EGDMA_{0.80}$)	11.9	14.5
[$Dod_{0.75}$/$PEG_{(17)0.25}$]-*p*($OEGMA_{50}$-*co*-$EGDMA_{0.80}$)	12.8	14.9
[$Dod_{0.50}$/$PEG_{(17)0.50}$]-*p*($OEGMA_{50}$-*co*-$EGDMA_{0.80}$)	14.5	15.1
[$Dod_{0.25}$/$PEG_{(17)0.75}$]-*p*($OEGMA_{50}$-*co*-$EGDMA_{0.80}$)	15.3	15.5

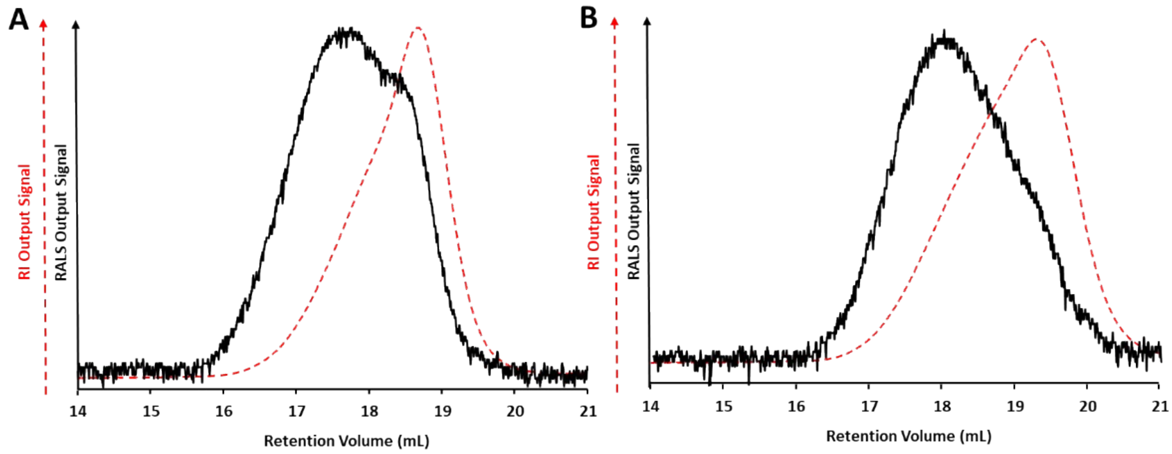

Figure S20 Comparisons of linear homopolymers and branched copolymers by TD-SEC at 60 °C in DMF/0.01 M LiBr. Overlays of refractive index (RI, red dashed lines) and right-angle light scattering (RALS, solid black lines) chromatograms for A) linear [$Xan_{1.00}$]-*p*($OEGMA_{50}$) and B) branched equivalent [$Xan_{1.00}$]-*p*($OEGMA_{50}$-*co*-$EGDMA_{0.80}$).

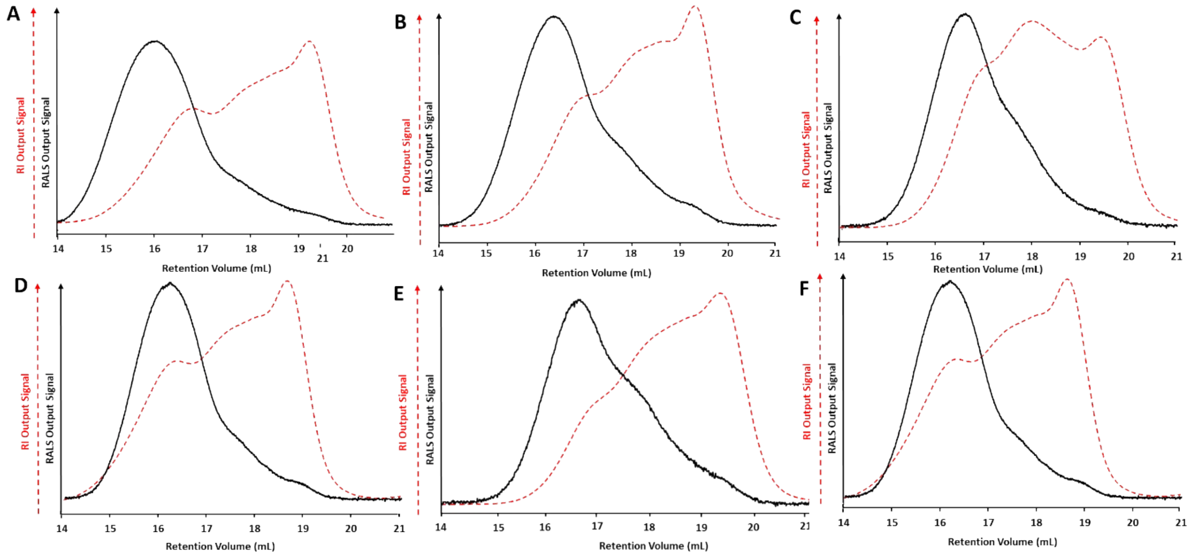

Figure S21 TD-SEC analysis of branched copolymers containing mixed chain-end functionality. Overlaid RI (dashed red lines) and RALS (solid black lines) chromatograms obtained from TD-SEC of Xan-Br initiated linear homopolymers and branched copolymers with the general composition [Xan$_y$/Dod$_x$]-*p*(OEGMA$_{50}$-*co*-EGDMA$_{0.80}$), where x + y = 1.00. A) linear homopolymer [Xan$_{1.00}$]-*p*(OEGMA$_{50}$) B) [Xan$_{0.08}$/Dod$_{0.92}$]-*p*(OEGMA$_{50}$-*co*-EGDMA$_{0.80}$), C) [Xan$_{0.25}$/Dod$_{0.75}$]-*p*(OEGMA$_{50}$-*co*-EGDMA$_{0.80}$), D) [Xan$_{0.50}$/Dod$_{0.50}$]-*p*(OEGMA$_{50}$-*co*-EGDMA$_{0.80}$), E) [Xan$_{0.75}$/Dod$_{0.25}$]-*p*(OEGMA$_{50}$-*co*-EGDMA$_{0.80}$), F) [Xan$_{0.90}$/Dod$_{0.10}$]-*p*(OEGMA$_{50}$-*co*-EGDMA$_{0.80}$) and G) [Xan$_{0.95}$/Dod$_{0.05}$]-*p*(OEGMA$_{50}$-*co*-EGDMA$_{0.80}$).

Table S6 Quantification of the contributions of high molecular weight species present within the molecular weight distributions to the overall mass of [Xan$_y$/Dod$_x$]-*p(*OEGMA$_{50}$-*co*-EGDMA$_{0.80}$) branched copolymers. TD-SEC analysis conducted at 60 °C using a DMF/0.01 M LiBr eluent.

**Branched copolymer**	**Weight %** **> 500 kg mol^{-1}**	**Weight %** **> 750 kg mol^{-1}**	**Weight %** **> 1, 000 kg mol^{-1}**
[Xan$_{0.08}$/Dod$_{0.92}$]-*p(*OEGMA$_{50}$-*co*-EGDMA$_{0.8}$)	17.7	11.6	8
[Xan$_{0.25}$/Dod$_{0.75}$]-*p(*OEGMA$_{50}$-*co*-EGDMA$_{0.8}$)	7.7	3.4	1.7
[Xan$_{0.50}$/Dod$_{0.50}$]-*p(*OEGMA$_{50}$-*co*-EGDMA$_{0.8}$)	18.9	13.0	9.4
[Xan$_{0.75}$/Dod$_{0.25}$]-*p(*OEGMA$_{50}$-*co*-EGDMA$_{0.8}$)	7.7	3.9	2.1
[Xan$_{0.90}$/Dod$_{0.10}$]-*p(*OEGMA$_{50}$-*co*-EGDMA$_{0.8}$)	5.6	2.1	0.9
[Xan$_{0.95}$/Dod$_{0.05}$]-*p(*OEGMA$_{50}$-*co*-EGDMA$_{0.8}$)	15.6	9.9	6.6

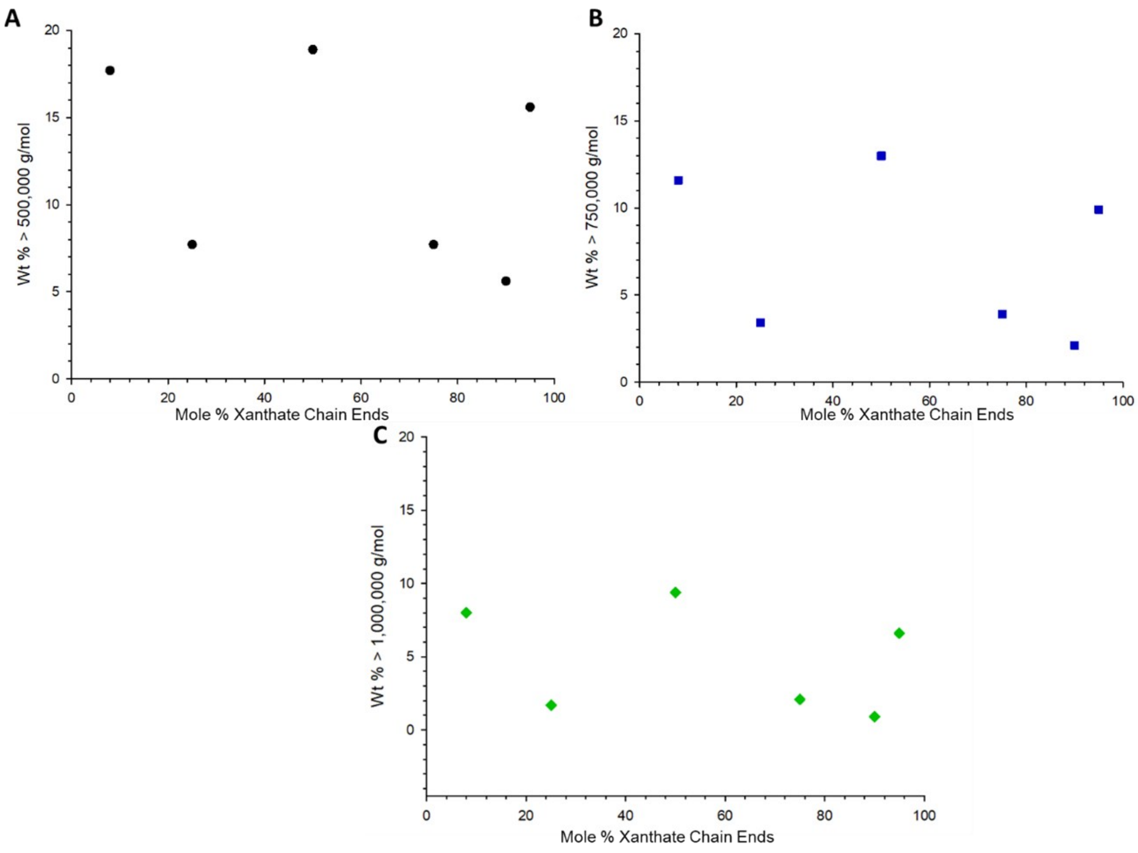

Figure S22 Variation in the weight percentage of high molecular weight species within branched copolymer molecular weight distributions across the [Xan_y/Dod_x]-*p(*$OEGMA_{50}$-*co*-$EGDMA_{0.80}$) series. Plots of the weight percent of branched copolymer species with molecular weights greater than A) 500,000 g mol^{-1} B) 750,000 g mol^{-1} and C) 1,000,000 g mol^{-1} against the molar percentage of Xan-Br initiator used in the copolymerisation.

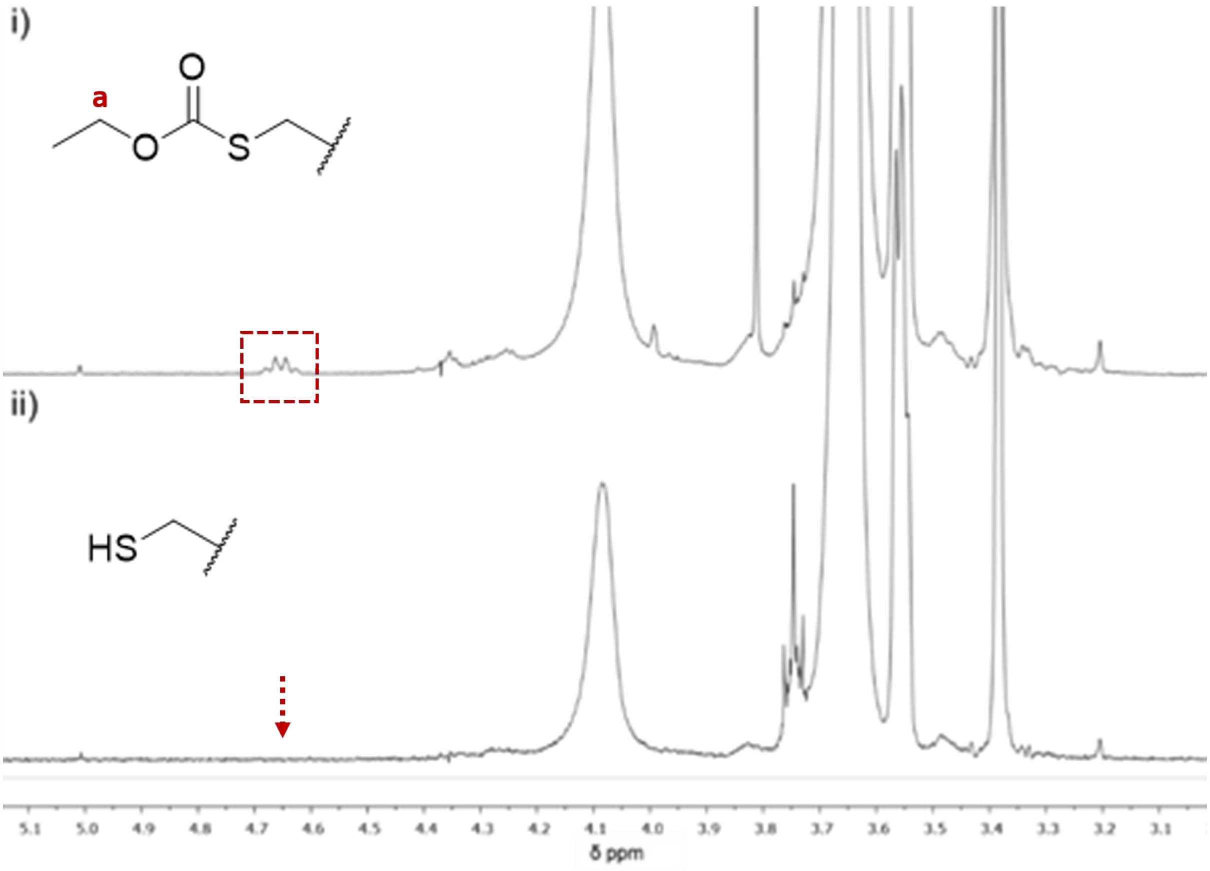

Figure S23 A) Confirmation of branched copolymer chain-end modification by ^{1}H NMR (400 MHz, $CDCl_3$). i) ^{1}H NMR spectrum of [$Xan_{0.75}$/$Dod_{0.25}$]-*p*($OEGMA_{50}$-*co*-$EGDMA_{0.80}$) prior to deprotection, showing the presence of the xanthate group at 4.66 ppm. ii) ^{1}H NMR spectrum of purified [$SH_{0.75}$/$Dod_{0.25}$]-*p*($OEGMA_{50}$-*co*-$EGDMA_{0.80}$ post deprotection, showing the absence of the xanthate group.

Table S7 Critical micelle concentrations (CMC) and minimum surface tension (ST_{min}) values determined for [Xan_y/Dod_x]-*p(*$OEGMA_{50}$-*co*-$EGDMA_{0.80}$) branched copolymers at 20 °C. Values are expressed as mg/L and errors are expressed as a graphical percentage error.

Branched Copolymer	Initial CMC (mg L^{-1})	ST_{min} (mg L^{-1})
[$SH_{0.25}$/$Dod_{0.75}$].*p*($OEGMA_{50}$-*co*-$EGDMA_{0.80}$)	9.12 x 10^{-6} (± 8.7 %)	1.46 x 10^{-4}
[$SH_{0.50}$/$Dod_{0.50}$].*p(*$OEGMA_{50}$-*co*-$EGDMA_{0.80}$)	9.13 x 10^{-6} (± 8.4 %)	1.50
[$SH_{0.75}$/$Dod_{0.25}$].*p(*$OEGMA_{50}$-*co*-$EGDMA_{0.80}$)	1.55 x 10^{-5} (± 16.2 %)	1.50
[$SH_{0.95}$/$Dod_{0.05}$].*p(*$OEGMA_{50}$-*co*-$EGDMA_{0.80}$)	1.83 x 10^{-5} (± 2.0 %)	1.50

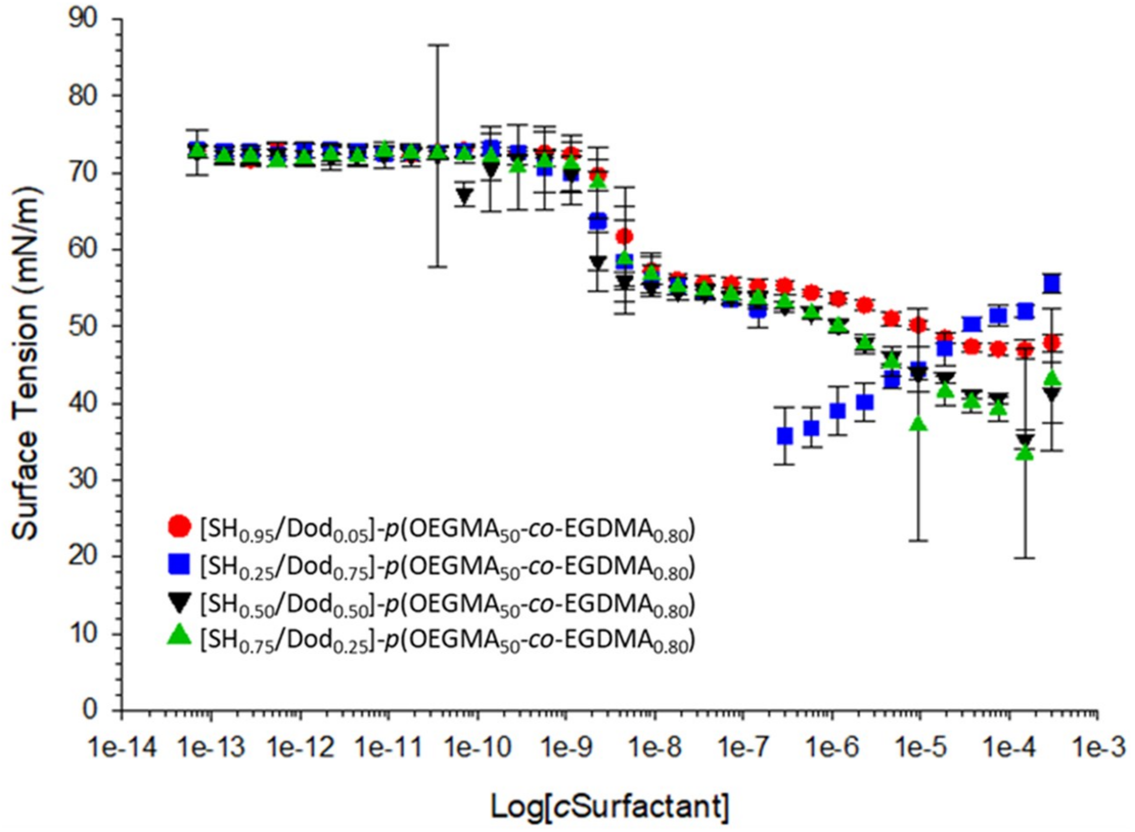

Figure S24 CMC behaviour of branched copolymers containing varied chain-end compositions. Overlaid plots of ST against branched copolymer concentration for: [$SH_{0.95}$/$Dod_{0.05}$]-*p*($OEGMA_{50}$-*co*-$EGDMA_{0.80}$) (red circles) [$SH_{0.75}$/$Dod_{0.25}$]-*p*($OEGMA_{50}$-*co*-$EGDMA_{0.80}$) (green triangles), [$SH_{0.50}$/$Dod_{0.50}$]-*p*($OEGMA_{50}$-*co*-$EGDMA_{0.80}$) (black downward triangles) and [$SH_{0.25}$/$Dod_{0.75}$]-*p*($OEGMA_{50}$-*co*-$EGDMA_{0.80}$) (blue squares).

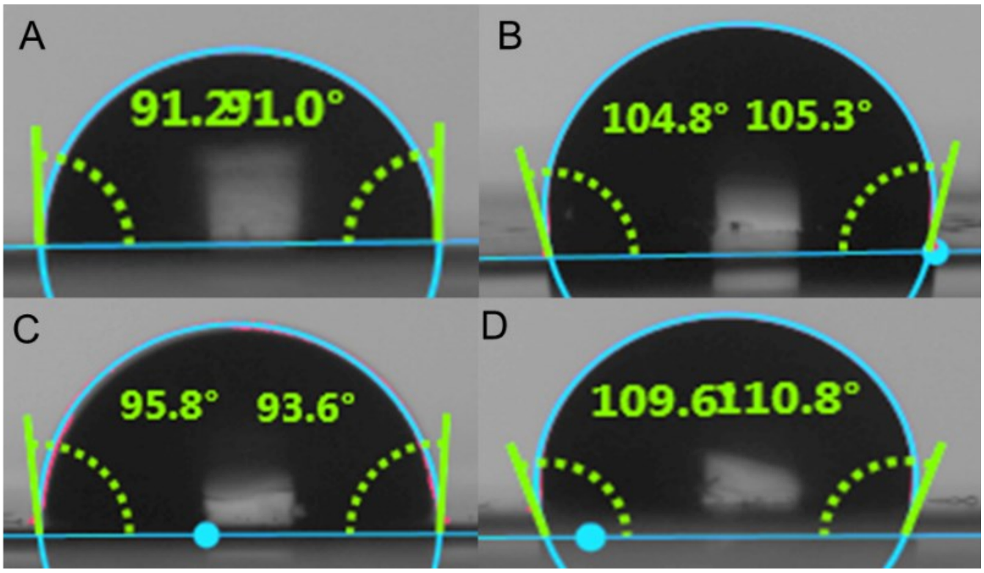

Figure S25 Contact angle measurements of droplets of aqueous branched copolymer solutions (5 wt %) resting on a PTFE substrate. A) [$SH_{0.05}$/$Dod_{0.95}$]-*p*($OEGMA_{50}$-*co*-$EGDMA_{0.80}$) B) [$SH_{0.25}$/$Dod_{0.75}$]-*p*($OEGMA_{50}$-*co*-$EGDMA_{0.80}$), C) [$SH_{0.50}$/$Dod_{0.50}$]-*p*($OEGMA_{50}$-*co*-$EGDMA_{0.80}$) and D) [$SH_{0.75}$/$Dod_{0.25}$]-*p*($OEGMA_{50}$-*co*-$EGDMA_{0.80}$).

Table S8 Contact angles measured for aqueous solutions of [Xan_y/Dod_x]-*p(*$OEGMA_{50}$-*co*-$EGDMA_{0.80}$) branched copolymers containing varied Xan and Dod chain-end compositions (5 wt %) on a PTFE surface.

**Polymer**	**Contact Angle (°)**		
	**Mean**	**Median**	**Std. Dev. (±)**
[$SH_{0.05}$/$Dod_{0.95}$]-*p*($OEGMA_{50}$-*co*-$EGDMA_{0.80}$)	85.28	85.03	6.39
[$SH_{0.25}$/$Dod_{0.75}$]-*p*($OEGMA_{50}$-*co*-$EGDMA_{0.80}$)	107.95	108.36	9.09
[$SH_{0.50}$/$Dod_{0.50}$]-*p*($OEGMA_{50}$-*co*-$EGDMA_{0.80}$)	93.71	93.62	6.66
[$SH_{0.75}$/$Dod_{0.25}$]-*p*($OEGMA_{50}$-*co*-$EGDMA_{0.80}$)	104.33	104.26	4.72

Table S9 Volume average diameters ($D_{[4,3]}$) of squalene-in-water emulsions over a 28-day time period.

	$D_{[4,3]}$ (μm)[a]			
**Polymer**	**Day 1**	**Day 14**	**Day 21**	**Day 28**
$[SH_{0.75}/Dod_{0.25}]$-*p*($OEGMA_{50}$-*co*-$EGDMA_{0.80}$)	15.4	15.8	15.9	16.2

[a] Values determined by laser diffraction.

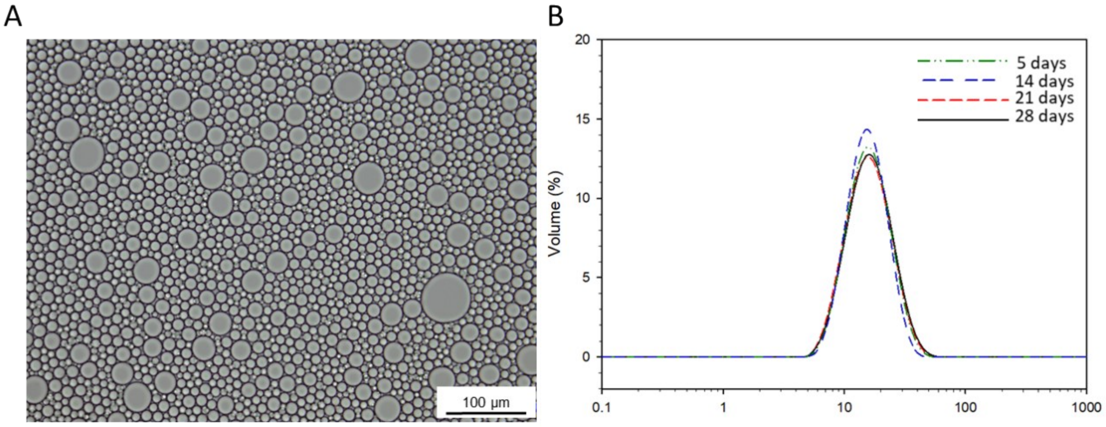

Figure S26 Analysis of the size, shape and stability of $[SH_{0.75}/Dod_{0.25}]$-*p*($OEGMA_{50}$-*co*-$EGDMA_{0.80}$)-stabilised squalene-in-water emulsions. A) Optical microscopy images of squalene-in-water emulsions 24 hours after emulsification. B) Analysis of the size ($D_{[4,3]}$) of squalene-in-water emulsion droplets by laser diffraction over a 28-day time period.

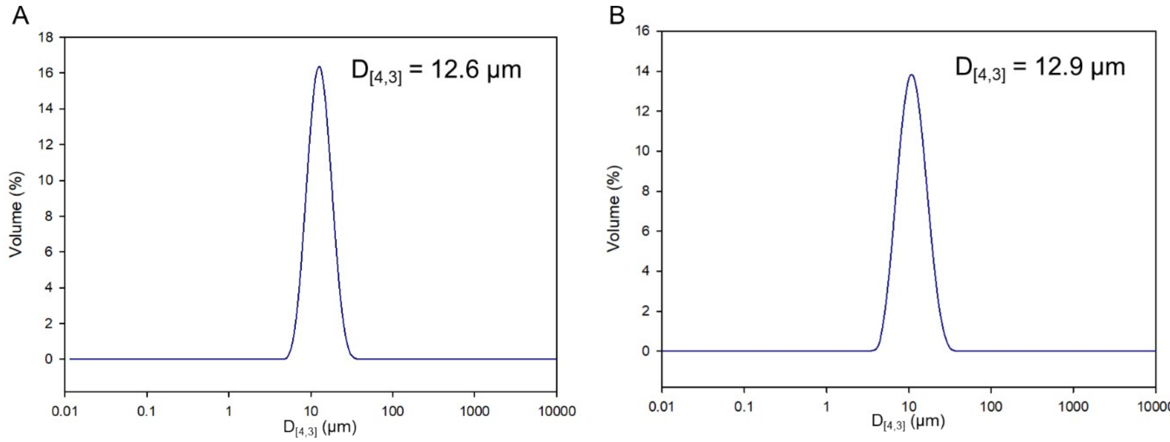

Figure S27 Emulsion droplet size distributions obtained by laser diffraction. A) $[SH_{0.75}/Dod_{0.25}]$-*p*($OEGMA_{50}$-*co*-$EGDMA_{0.80}$)-, B) $[Dod_{1.00}]$-*p*($OEGMA_{50}$-*co*-$EGDMA_{0.80}$)-stabilised squalene-in-water emulsions containing Oil Blue A (0.1 wt % w.r.t. oil phase).

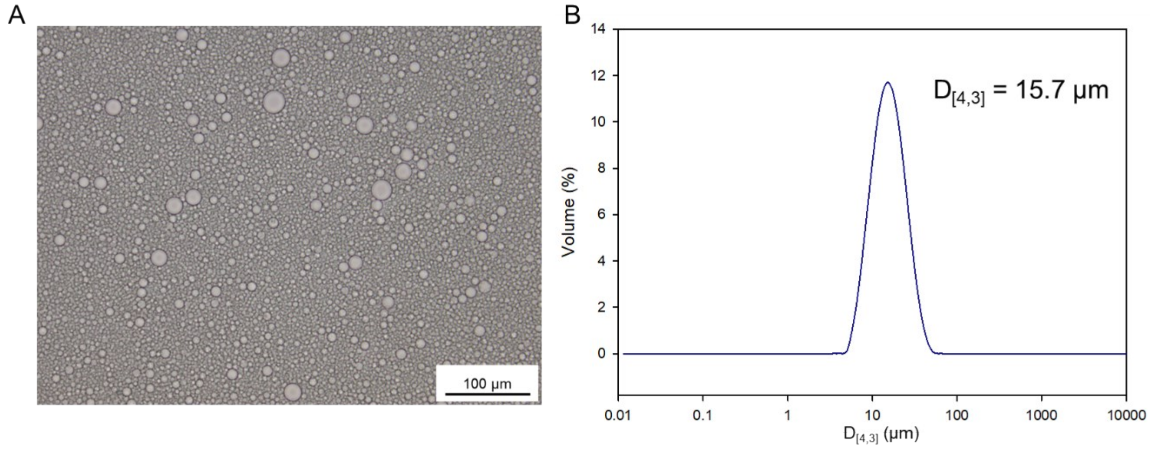

Figure S28 Oil Red O loaded (0.1 wt % w.r.t. oil phase) [$SH_{0.75}$/$Dod_{0.25}$]-*p*($OEGMA_{50}$-*co*-$EGDMA_{0.80}$)-stabilised squalene-in-water emulsions. A) Image obtained by optical microscopy. B) Emulsion droplet size distribution obtained by laser diffraction.
